# Disrupted terminal bilirubin catabolism links *Lachnospiraceae* depletion to inflammatory bowel disease

**DOI:** 10.3389/fmicb.2026.1871609

**Published:** 2026-08-17

**Authors:** Omer Sabti, Ksenia Lialin-Tzadikov, Valeria Ivanova, Mally Dori-Bachash, Shaked Uzi-Gavrilov, Zohar Tik, Roi Mashiach, Alvah Zorea, Itzhak Mizrahi, Arik Segal, Keren Moyal-Attias, Eran Elinav, Michael M. Meijler

**Affiliations:** 1 Department of Chemistry, Ben Gurion University of the Negev, Be’er Sheva, Israel; 2 Department of Systems Immunology, Weizmann Institute of Science, Rehovot, Israel; 3 Department of Life Sciences, Ben Gurion University of the Negev, Be’er Sheva, Israel; 4 Department of Gastroenterology, Soroka University Medical Center, Be’er Sheva, Israel; 5 Division of Microbiome and Cancer, German Cancer Research Center (DKFZ), Heidelberg, Germany

**Keywords:** bilirubin, IBD, metabolomics, microbial chemical communication, microbiota

## Abstract

**Background:**

The gut microbiome mediates the reductive catabolism of bilirubin into urobilinoids, yet the bacteria and enzymes responsible for the later steps of this pathway remain largely unknown.

**Methods:**

Here, we combine untargeted UPLC/HRMS metabolomics with shotgun metagenomic sequencing of fecal samples from 119 participants, including patients with Crohn’s disease, ulcerative colitis, and healthy controls, to map disruptions in the bilirubin catabolic pathway in inflammatory bowel disease (IBD).

**Results:**

We show that stercobilinogen and stercobilin, the terminal metabolites of this pathway, are depleted (*p* < 0.01) in IBD patients irrespective of disease subtype, while upstream intermediates, D-urobilinogen, remain unchanged. This metabolic bottleneck coincides with a marked reduction in members of the *Lachnospiraceae* family, specifically *Blautia* sp. SG-772 and three uncharacterized species, which show strong positive correlations with stercobilinogen and stercobilin levels.

**Discussion:**

These findings implicate *Lachnospiraceae* as key mediators of the yet unknown enzymatic conversion of I-urobilinogen to stercobilinogen, extending the family’s known metabolic repertoire beyond short-chain fatty acid production. Given that bilirubin and its reduced metabolite stercobilinogen possess antioxidant properties, their depletion may contribute to the oxidative burden in the IBD gut. Conversely, the concurrent elevation of D-urobilin, which lacks these protective properties and has been linked to metabolic dysfunction in other contexts, may further exacerbate inflammation. Our results identify fecal bilirubin metabolites as candidate biomarkers of microbial dysbiosis in IBD and nominate specific *Lachnospiraceae* taxa for functional characterization of the missing stercobilinogen reductase.

## 1 Introduction

The complex interdependence between the human body and its diverse microbial inhabitants mutually influences human cells and microbes (Foster and McVey Neufeld, 2013; Gregor et al., 2022). This is particularly evident in digestion and nutrient absorption, especially within the gut (Belkaid and Hand, 2014; Lee et al., 2024). Imbalances in this intricate relationship can trigger and affect conditions such as inflammatory bowel diseases (IBDs), including Crohn’s disease (CD) and ulcerative colitis (UC) (Jacobs et al., 2016). The relationship between the gut microbiome and IBD is complex, with much still to be understood. It’s well established that IBD is strongly associated with dysbiosis of the gut microbiome, a measurable shift in bacterial diversity, richness, and composition compared with healthy individuals (Abdelbary et al., 2022; Belkaid and Hand, 2014; Ma et al., 2022; Rapozo et al., 2017; Wright et al., 2015).

Active disease is characterized by reduced bacterial diversity and a microbiome imbalance, with fewer beneficial bacteria, such as some Firmicutes, and more potentially harmful species (Rashed et al., 2022; Wright et al., 2015). This connection also involves metabolic pathways; for instance, altered bile acid metabolism and shifts in the bacterial populations that transform them have been implicated in CD (Fogelson et al., 2023; Kumar et al., 2022). The gut microbiota modulates bile acid profiles by converting primary bile acids, synthesized by the liver, into secondary bile acids (Fogelson et al., 2023; Lee et al., 2024; Mohanty et al., 2024). However, a fundamental unanswered question is whether these shifts in the gut microbiome are a cause of IBD or simply a consequence of the inflammation, as evidence supports both possibilities without establishing clear causality in humans (Haag and Siegmund, 2015; Ma et al., 2022; Zoroddu et al., 2024). Recent prospective studies have begun to elucidate this timeline. For instance, Xue et al. identified specific serum metabolic signatures, such as elevated quinolinate and reduced ascorbate, that precede the diagnosis of Crohn’s disease by years, suggesting that metabolic dysregulation is an early driver of pathogenesis rather than solely a consequence (Xue et al., 2025). Furthermore, while broad patterns, such as the loss of diversity, are recognized, the specific microbial species, strains, or community interactions that either drive or protect against the disease remain uncertain and may vary across different populations (Chen et al., 2024; Ma et al., 2022). A deeper understanding of the dynamics underlying healthy and abnormal conditions is needed to better comprehend human-microbial symbiosis and dysbiosis.

Vast communication networks exist within the gut, connecting the host to the gut microbiome through the secretion and sensing of small molecules, collectively referred to as the gut metabolome (Fogelson et al., 2023; Gregor et al., 2022; Levhar et al., 2026). We decided to explore previously unknown molecular links between microbiota dynamics and host gut health, using untargeted metabolomics as our primary tool. We used ultra-performance liquid chromatography/high-resolution mass spectrometry (UPLC/HRMS) to comprehensively analyse metabolomes from large cohorts of fecal matter samples, obtained from both Crohn’s and colitis patients, as well as healthy volunteers. Multivariate data analysis, combined with feature-based molecular networking and other tools within the community-based online platform Global Natural Product Social Molecular Networking (GNPS), enabled the identification of several compound classes, the most significant of which is a cluster of bilirubin-like molecules (Nothias et al., 2020; Wang et al., 2016).

**FIGURE 1:**
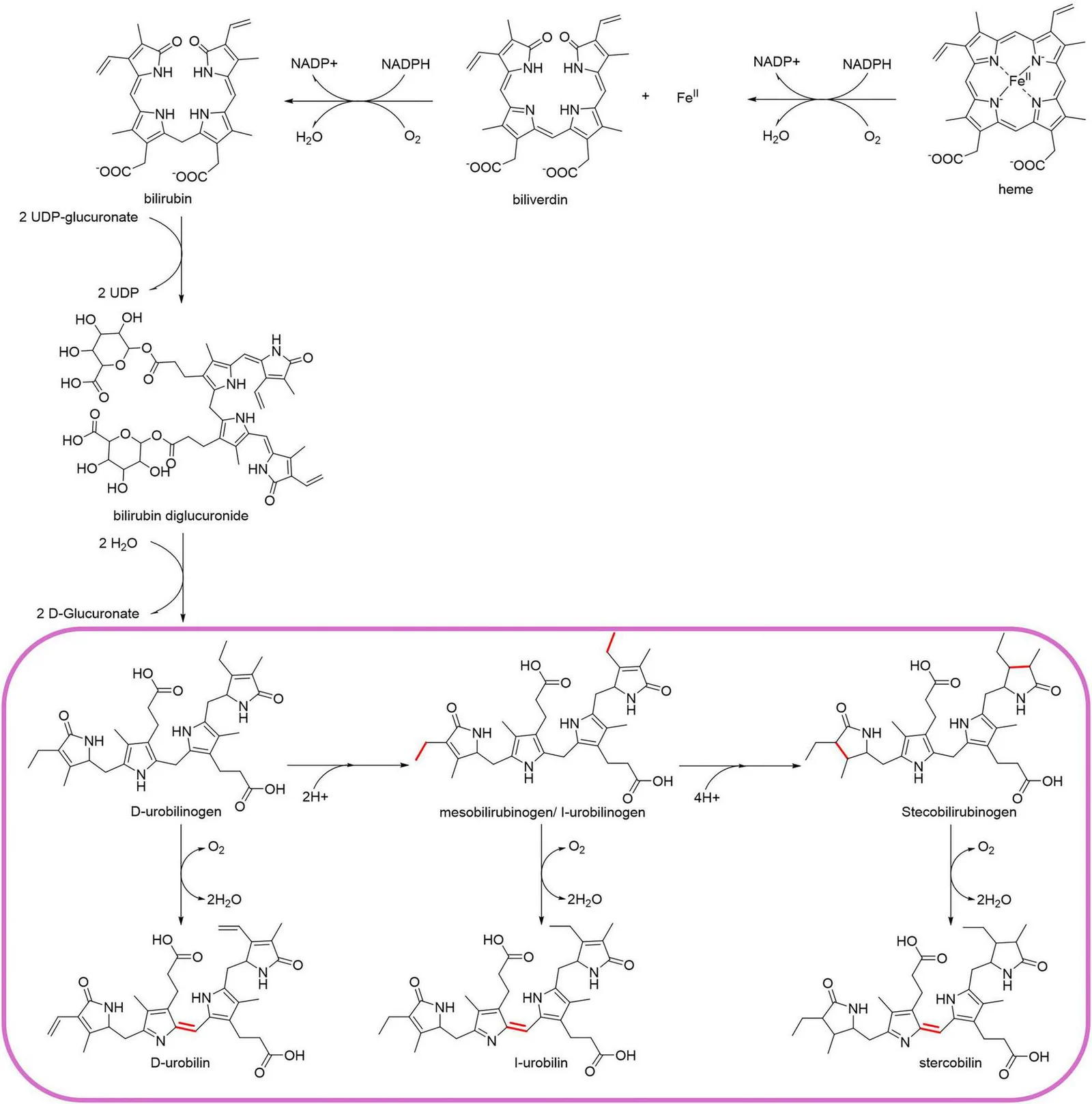
A chemical representation of the heme’s catabolic pathway. The steps outlined in the box occur in the gut by members of the local microbiome. Changes in the chemical structure of bilirubin’s downstream metabolites are highlighted.

Serum bilirubin, a yellow pigment derived from heme degradation, is a key metabolite with potential anti-inflammatory effects in IBD, as it protects against oxidative stress (Blanco and Blanco, 2017; Zoroddu et al., 2024). This substance is the second product formed during heme catabolism and is known for its antioxidant properties (Canesin et al., 2020; Zhang et al., 2025). Heme, the iron-containing non-protein component of hemoglobin, undergoes catabolism in which heme oxygenase breaks down the porphyrin ring, resulting in biliverdin, carbon monoxide, and free iron. Subsequently, biliverdin reductase converts it into unconjugated bilirubin, which circulates in the bloodstream bound to albumin due to its hydrophobic nature. Hepatocytes absorb bilirubin through organic anion transporters, where UDP-glucuronosyltransferase 1A1 conjugates it with glucuronic acid, making it water-soluble for biliary excretion (Blanco and Blanco, 2017; Engelking, 2015). Microbial β-glucuronidases break down bilirubin glucuronides in the intestines, enabling the gut microbiome to convert them into urobilinoids through various redox reactions (Boyle et al., 2026, 2026; Hall et al., 2024). These urobilinoids include D-urobilinogen, D-urobilin, I-urobilinogen, I-urobilin, stercobilinogen, and stercobilin. While most urobilinogen is oxidized to stercobilin for fecal excretion, roughly 15% is recycled through the enterohepatic pathway before being eliminated by the kidneys as urobilin (Figure 1; Engelking, 2015). Consequently, the gut microbiome plays a seminal role in regulating the impact of urobilinoids on the colon (Canesin et al., 2020; Mölzer et al., 2017). Beyond the gut, elevated urobilin levels have been linked to increased adiposity and insulin resistance in humans, highlighting its connection to systemic inflammation and metabolic dysfunction (Kipp et al., 2023). This is particularly significant for digestion and nutrient absorption, especially in the gut. A meta-analysis by Zoroddu, Stefano et al. found that serum bilirubin concentrations are lower in IBD patients than in healthy controls (HC). Therefore, serum bilirubin and its gut metabolites may be potential biomarkers in IBD (Zoroddu et al., 2024).

A recent study by Hall et al. found that BilR, a bilirubin-reducing enzyme primarily encoded by the gut microbiome and predominantly belonging to the Firmicutes, is present at lower levels in patients with IBD (Hall et al., 2024). This decrease in BilR levels indicates a disruption in the gut microbiome’s ability to process bilirubin in patients with IBD. Such a reduction may lead to changes in bilirubin metabolism, potentially worsening intestinal inflammation and oxidative stress due to a diminished conversion of harmful bilirubin into more easily excretable urobilinoids. However, the specific enzyme responsible for the next step in the conversion of I-urobilinogen to stercobilinogen remains unidentified. Therefore, we set out to examine in detail how factors in the gut bilirubin catabolic pathway may play a role in the disease state in IBDs and whether they could serve as biomarkers.

## 2 Materials and methods

### 2.1 Study population

In this cohort study, three groups of adult participants were enrolled (aged ≥ 18 years), UC (*n* = 31), CD (*n* = 39), and healthy individuals (*n* = 49) as a control group. A healthy individual was marked as such if they did not report having a GI disorder. Detailed information regarding the participants’ demographics, lifestyle, and disease state was also collected, including age, sex, smoking habits, diet, alcohol consumption, supplements, disease severity, and current and past medical and surgical treatments. Inclusion criteria were adults ( ≥ 18 years) with a confirmed IBD diagnosis or healthy volunteers without GI disease. Exclusion criteria included antibiotic use within 4 weeks of collection, active gastrointestinal infection, or inability to provide informed consent. All participants provided written informed consent. The study was approved by the Soroka Helsinki Committee for the Protection of Human Subjects (#0148-15-SOR, 2/7/2021).

#### 2.1.1 Cohort characteristics

Clinical data analysis was performed in RStudio. ANOVA test and the Chi-square test of independence for the respective variables generated *P*-values. Raw data are available in Supplementary Table 1.

#### 2.1.2 Sample collection

Fecal samples were collected from patients at the Department of Gastroenterology at Soroka Hospital and from healthy volunteers at Ben Gurion University. They were stored in 50 mL Falcon tubes at −80°C until further treatment.

### 2.2 Metabolomics

#### 2.2.1 Metabolite extraction

Collected fecal samples underwent untargeted metabolomics using liquid chromatography-tandem mass spectrometry (LC-MS/MS). A small amount from each sample was transferred to an Eppendorf tube for analysis. The new samples (i.e., in the Eppendorf tube) were frozen in liquid nitrogen and then freeze-dried for 24 h using a lyophilizer. The dry matter was weighed, suspended in cold MeOH/H2O (1:1 v/v) at 49 mg/mL, and stirred for half a minute. After that, the samples were loaded into a centrifuge and spun at 4°C for 10 min at 10,000 *g*. The supernatant was collected, and a mixed sample was used as a quality control (QC).

#### 2.2.2 UHPLC-HRMS/MS analysis

The samples were examined by using an UltiMate 3000 UHPLC+ focused LC-MS system (Thermo Scientific™) with 2.6 μm (100 * 2.1 mm) C18 Accucore™ UHPLC column (Thermo Scientific™), followed by Q Exactive™ Focus Hybrid Quadrupole-Orbitrap Mass Spectrometer (Thermo Scientific™) analysis. The run conditions were a flow rate of 0.4 mL/min, with the composition of solvent A being 99.9% water and 0.1% formic acid, solvent B being 99.9% acetonitrile, and 0.1% formic acid. The gradients started with 90%:10% A:B for 2 min. After 13 min, the gradient increased to 10%:90% A:B and stayed in this composition until 17 min. The gradient changed to 90%:10% A:B until 20 min and was maintained until 22 min. The QC was added between every ten samples. For full MS^1^ spectra acquisition, the instrument was set to capture features from m/z 100–1000 Da in positive ion mode at 70,000 resolutions. For MS^2^ data acquisition, the three most intense precursor ions per MS^1^ scan were selected for MS^2^ fragmentation at 17,500 resolution.

#### 2.2.3 Data processing

All MS data were analyzed using Freestyle and Xcalibur software (Thermo Scientific™) (Supplementary Figure 3). Following the initial examination, the raw files were centroided and converted to.mzML files using the open-source program MSconvert9 (Adusumilli and Mallick, 2017). Then, all files were processed using MZmine 3.9.0 (Schmid et al., 2023). For a Feature-Based Molecular Network and MolNetEnhancer analysis in the Global Natural Product Social Molecular Networking (GNPS)^1^, the quantification table in MGF format from MZmine, the metadata file, and the original.mzML files were uploaded to GNPS via WinSCP (Aron et al., 2020). The FBMN was generated with a precursor mass tolerance of 0.02 Da, a fragment mass tolerance of 0.02 Da, a minimum cosine similarity of 0.7, and a minimum of 6 matched peaks. It was visualized in Cytoscape using the GNPS format (Nothias et al., 2020).

For statistical analysis and group comparisons, the quantification table output from Mzmine 3.9.0, in CSV format, was used. Data cleaning and processing were performed using RStudio. Features were filtered based on blank-to-sample intensity ratios. Missing values were imputed with random values between 1 and the calculated Limit of Detection (LOD). Following imputation, the data were normalized using the Total Ion Current (TIC) method via the “Kodama” package. Finally, the processed data were log-transformed and scaled prior to PCoA. The ANOVA test and comparison visualization were performed using GraphPad Prism 9.

#### 2.2.4 I-urobilinogen validation

For validation, a mixed sample (i.e., QC) was compared to a standard. The standard was purchased from Medix Biochemica. UPLCA-HRMS analysis was conducted as described in the previous section. The standard was run twice, once without oxygen exposure and once with oxygen exposure. The validation focused on the mass at 592.3261 m/z (Supplementary Figures 3–6).

### 2.3 Metagenomics

#### 2.3.1 DNA purification and shotgun sequencing

Fecal DNA samples were extracted and purified using a Purelink Microbiome DNA purification kit (Invitrogen). For shotgun sequencing, Illumina libraries were prepared using the Nextera DNA Sample Prep kit (Illumina, 20034198) according to the manufacturer’s protocol, and sequenced on the Illumina Novaseq platform with a read length of 122 base pairs (bp).

QC trimming of raw metagenomic reads was performed using fastp, version 0.22.0 (Chen et al., 2018), with a minimum read length set to 50 bp. Host sequences were removed by aligning the reads to the human reference genome (GRCh37) using Bowtie2, version 2.4.5 (Langmead and Salzberg, 2012). Unmapped reads were retained using SAMtools, version 1.15 (Li et al., 2009). The cleaned reads were then subsampled to 1,000,000 reads per sample using Seqtk, version 1.3^2^. For the taxonomic assignment of bacterial DNA, exact k-mer alignment was conducted using Kraken2, version 2.1.2 (Wood et al., 2019) against the Genome Taxonomy Database (GTDB) (version 95)^3^. This process was supplemented with human and mouse reference genomes to minimize false taxonomic annotations stemming from residual host reads. Species-level bacterial abundance was further refined through Bayesian re-estimation using Bracken, version 2.7 (Lu et al., 2017), resulting in a bacterial relative abundance table (Supplementary Table 2). The entire workflow was managed using Snakemake, version 7.12.1 (Mölder et al., 2021).

#### 2.3.2 Data processing

The statistical analyses for metagenomics were based on the bacterial relative abundance table (Supplementary Table 2), and all the analyses and visualizations were performed in RStudio using the “tidyverse,” “vegan,” “phyloseq,” “ggpubr,” and “tidygraph” packages. For microbiome community analysis, bacterial abundance data were processed to calculate relative abundances or to log-transform them for multivariate analyses. Alpha diversity was evaluated using the Shannon, Simpson, Observed, Chao1, and ACE indices. Differences in alpha diversity and bacterial abundance between groups (HC, CD, UC) were assessed using the Kruskal-Wallis test or one-way ANOVA, followed by pairwise Wilcoxon or Dunn’s *post-hoc* tests with Bonferroni correction for multiple comparisons.

Beta diversity was characterized using Bray-Curtis, Jaccard, and Euclidean dissimilarity matrices. Community composition differences were visualized using Principal Coordinates Analysis (PCoA). The statistical significance of between-group separation was assessed using Permutational Multivariate Analysis of Variance (PERMANOVA) and Analysis of Similarities (ANOSIM).

**FIGURE 2:**
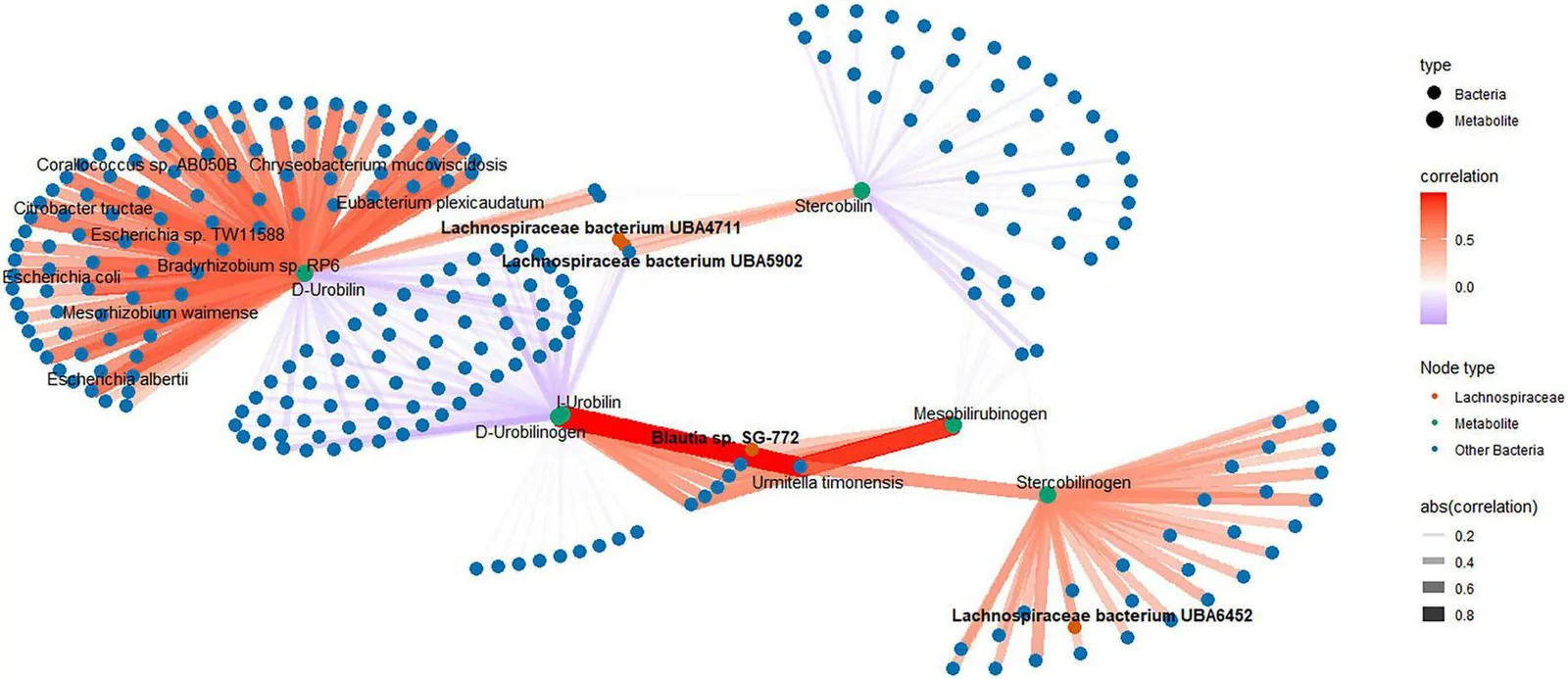
Correlation network of bacterial taxa and bilirubin downstream metabolites. Nodes represent bacteria (red) and metabolites (blue). Edges represent correlations, with color indicating direction (red for positive, purple for negative) and thickness showing absolute strength.

For multi-omics integration, metabolite features corresponding to bilirubin analogs were merged using a 10 ppm mass tolerance and a 0.5 min retention time window. Pearson correlation coefficients were calculated between the abundances of all 1,621 bacterial species identified in the metagenomics dataset and the intensities of each of the six bilirubin metabolites across common samples (total pairwise tests = 9,726). Of these, 472 species exhibited at least one nominally significant association (*p* < 0.05). Benjamini–Hochberg correction was applied to this subset. For visualization in Figure 2, edges were retained only if | r| > 0.5 and the BH-adjusted *p*-value was q < 0.05. All depicted associations satisfy both criteria and are displayed using the Kamada-Kawai layout algorithm.

## 3 Results

### 3.1 Cohort characteristics

The study population comprised 119 participants, UC (*n* = 31), CD (*n* = 39), and HC (*n* = 49), with similar mean ages across the three groups (mean age range: 34.2–36.8 years, *p* = 0.42). Six samples were excluded due to being too small or because of other underlying health conditions. Disease duration was comparable between UC and CD groups (8.2 ± 6.4 and 9.1 ± 7.2 years, respectively, *p* = 0.56). IBD patient groups showed significantly higher rates of dietary modifications (UC: 48.4%, CD: 56.4% vs. controls: 24.5%, *p* < 0.01). Supplement use was also markedly higher in IBD patients, with vitamin D and iron supplementation being significantly more common in both UC and CD groups than in HC (*p* < 0.01) (Table 1).

**TABLE 1:** Demographic and clinical characteristics.

Category	Characteristic	HC (*n* = 49)	UC (*n* = 31)	CD (*n* = 39)	*P*-value
Demographics	Age (years; mean ± SD)	34.2 ± 9.8	36.5 ± 11.2	36.8 ± 10.5	0.42
	Age range	19–58	22–70	20–65	–
	Male *n* (%)	25 (51.0)	16 (51.6)	13 (33.3)	0.18
	Female *n* (%)	24 (49.0)	15 (48.4)	26 (66.7)	0.78
Clinical characteristics	Disease duration (years; mean ± SD)	N/A	8.2 ± 6.4	9.1 ± 7.2	0.56
	Current smoker *n* (%)	7 (14.3)	3 (9.7)	5 (12.8)	0.84
Disease activity	Remission n/n-with-data (%)	N/A	13/24 (54.5)	7/38 (18.4)	0.03
	Calprotectin μg/g (mean ± SD)	N/A	285 ± 156	312 ± 178	0.48
Medications	Any IBD medication *n* (%)	N/A	28 (90.3)	35 (89.7)	0.93
	Biologics *n* (%)	N/A	12 (38.7)	18 (46.2)	0.54
	Immunomodulators *n* (%)	N/A	15 (48.4)	21 (53.8)	0.65
	5-ASA *n* (%)	N/A	22 (71.0)	15 (38.5)	< 0.01

Data presented as mean ± standard deviation. N/A, not applicable. *P*-values were calculated using the Chi-square test for categorical variables and Student’s *t-*test or ANOVA for continuous variables as appropriate. Additional data are listed in Supplementary Tables 1, 1a.

### 3.2 Metagenomics profile

**FIGURE 3:**
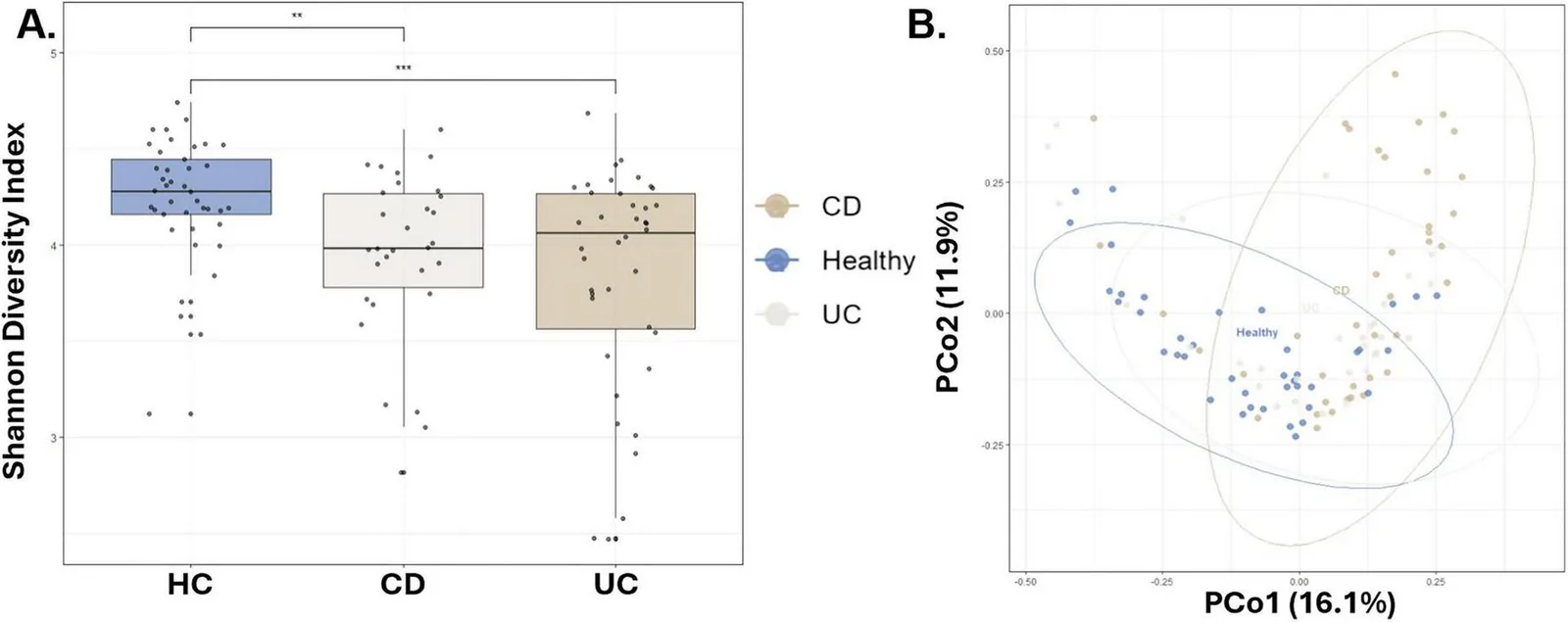
Experimental results of shotgun sequencing from the stool samples. **(A)** Box plot illustrating the alpha diversity index for each group. **P < 0.01, ***P < 0.001; **(B)** Beta diversity analysis of microbial communities, visualized using Principal Coordinates Analysis (PCoA) based on Bray-Curtis dissimilarity.

A total of 1,621 different microbial species were observed in the samples. The abundance of each identified strain was calculated based on the sample, and all further analyses relied on Supplementary Table 2. The alpha-diversity analysis (Shannon index) showed a slight decrease in population diversity among patients (Figure 3A). The patient groups were categorized according to their active disease and remission status. The results indicated no difference in microbial diversity between patients in remission and HC (Supplementary Figure 1). Meanwhile, individuals with active disease showed a notable reduction in gut bacterial diversity. The beta diversity of the microbiome community is illustrated in a PCoA plot (Figure 3B), indicating that the compositions of the two patient groups were similar (Supplementary Figure 7). Despite similarities between the HC and patient groups, a noticeable difference existed. The changes in abundance are displayed in Supplementary Figure 8. For all species represented in the figure, the *p*-value is < 0.01 or smaller. As mentioned, 1,621 species were initially observed, but only 472 were found to have changed significantly (*p*-value < 0.05) in patient samples compared with HC.

### 3.3 Metabolomics profile

**FIGURE 4:**
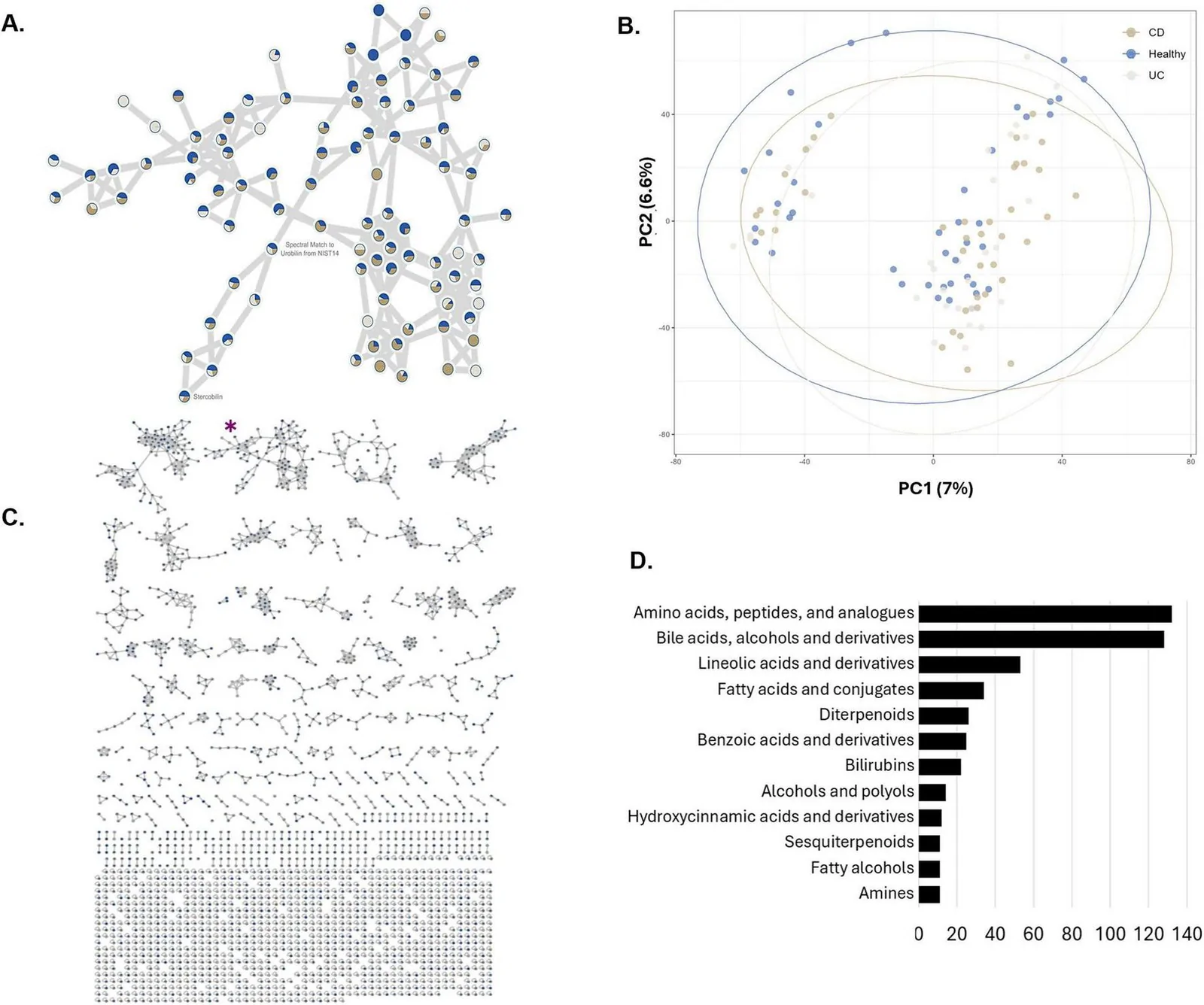
**(A)** Cluster of bilirubin-related metabolites. **(B)** PCoA showing overall metabolic differences among groups. **(C)** Feature-Based Molecular Networking was created by GNPS. Each node represents a metabolite in the samples, and an edge between two nodes indicates that the metabolites share a similar structure. An asterisk highlights the bilirubin cluster shown in panel **(A)**. **(D)** The number of metabolites that were identified as belonging to a chemical family based on MolNetEnhancer results.

**FIGURE 5:**
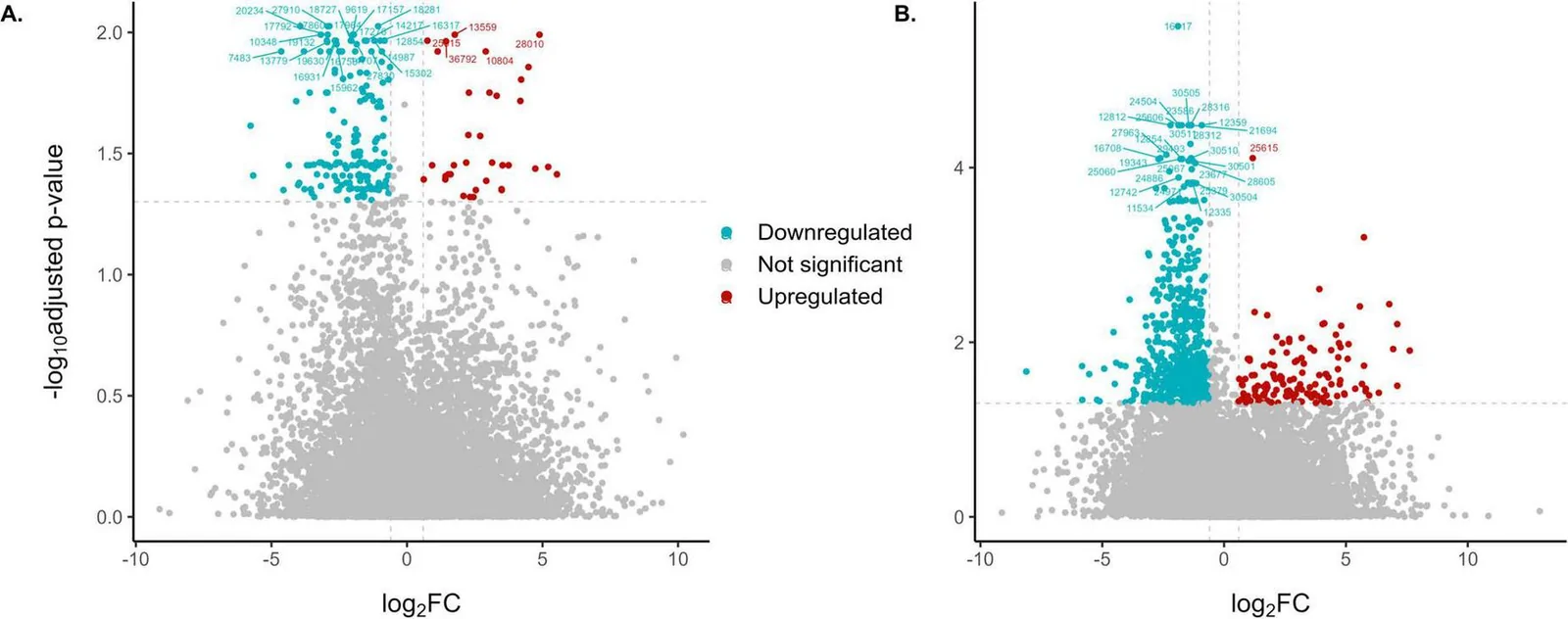
Volcano plots showing upregulated and downregulated metabolites in the patient groups compared to HC. Thresholds were p-adj = 0.05 and FC = 0.6. For the Top 50 metabolites, the feature ID is shown. **(A)** HC vs UC. **(B)** HC vs CD.

A sample’s quality control (QC) examination showed a pattern in patient groups (Supplementary Figure 2), compared to the HC group. However, the PCoA for the group’s metabolic profiles did not reveal significant differences (Figure 4B). In contrast, a volcano plot comparing the control and patient groups showed more than 100 features that were either upregulated or downregulated (Figure 5). The FBMN analysis revealed multiple clusters of compounds with structure similarities (Figure 4C). A small portion of these features was annotated using the MS libraries available on the GNPS platform. In the highlighted cluster, urobilin and stercobilin, which are bilirubin’s metabolites, were annotated (Figure 4A). Using this FBMN as a base, another computational analysis feature, MolNetEnhancer, was conducted (Figure 4D; Ernst et al., 2019). Most annotated metabolites were associated with amino acids and peptides, while bile acids accounted for the second most common subclass.

### 3.4 Bilirubin metabolites

Before examining the relationships among different metabolites and their relative amounts in each group, we validated the identification the bilirubin metabolites according to the Metabolomics Standards Initiative (MSI) (Fiehn et al., 2007). I-urobilinogen (also known as mesobilirubinogen), a key element in this pathway, was validated at MSI level 1, which is identification confirmed with an authentic reference standard using matching retention time, accurate mass, and MS/MS fragmentation, and D-urobilinogen, stercobilinogen, D-urobilin, I-urobilin (known as urobilin), and stercobilin at MSI Level 2 which is putative identification based on spectral library matching without an authentic standard. The level 2 metabolites were validated by comparing the MS^2^ spectral features with those in the GNPS libraries and a custom-made SIRIUS library (Dührkop et al., 2019). Authentic standards for the level 2 metabolites were not commercially available at the time of study, precluding level 1 confirmation of these metabolites.

**FIGURE 6:**
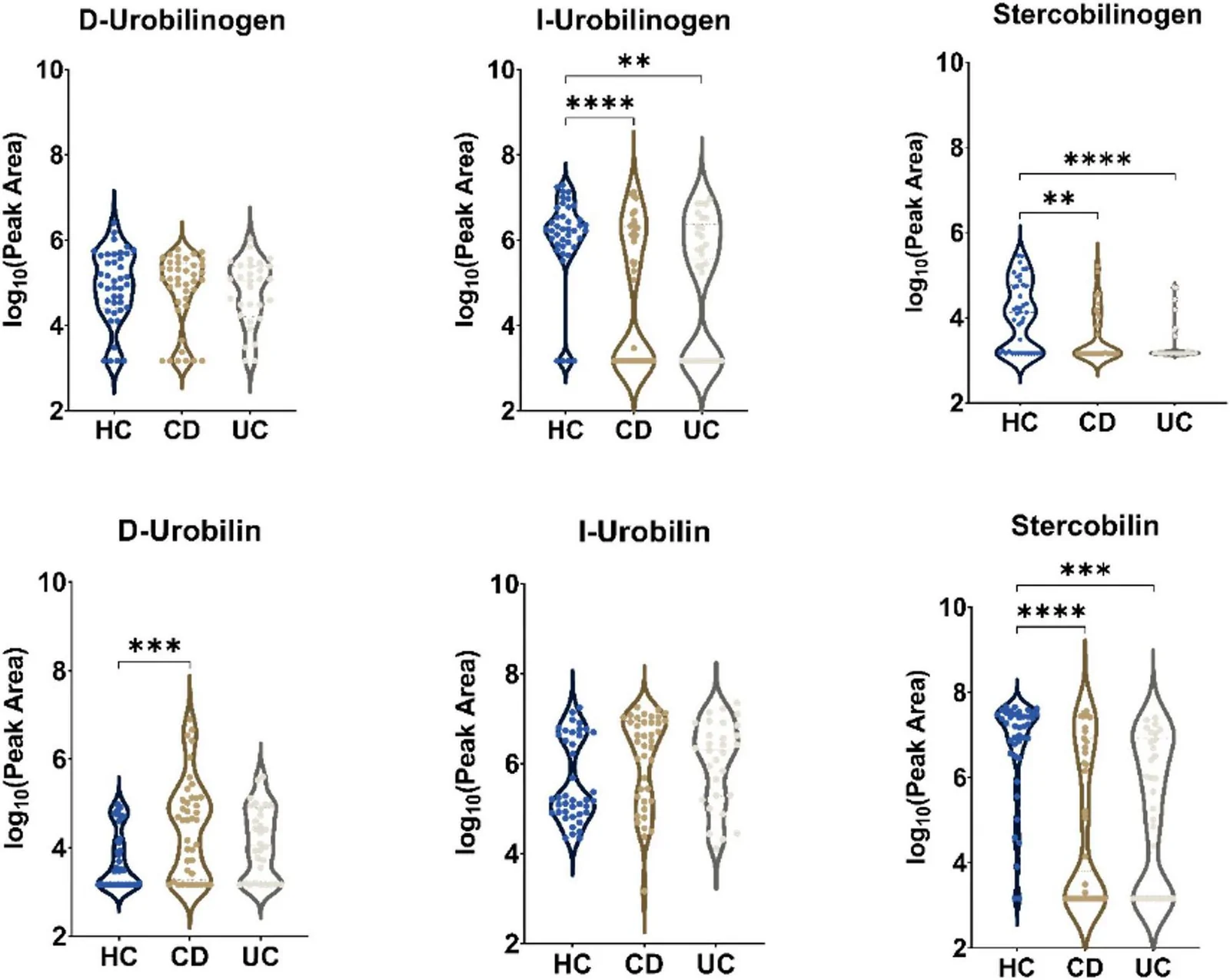
Relative abundance of selected metabolites across the cohort groups. Each violin plot shows the log10 of the peak area (ion count) for a distinct bilirubin metabolite. **P < 0.01, ***P < 0.001, and ****P < 0.0001.

Following validation, we conducted an ANOVA to determine whether there were significant differences in the relative amounts of bilirubin metabolites across samples. The results indicate varying concentrations of bilirubin metabolites across the study groups (Figure 6). While D-urobilinogen levels were similar across all groups, I-urobilinogen concentrations were significantly higher in HC than in CD and UC patients (*p* < 0.01 and *p* < 0.0001, respectively). Both stercobilinogen and its oxidized form, stercobilin, showed notably lower levels in IBD patients than in HC. D-urobilin levels were higher in CD patients than in HC, whereas I-urobilin concentrations were comparable across all groups.

### 3.5 Multi-omics correlations

**FIGURE 7:**
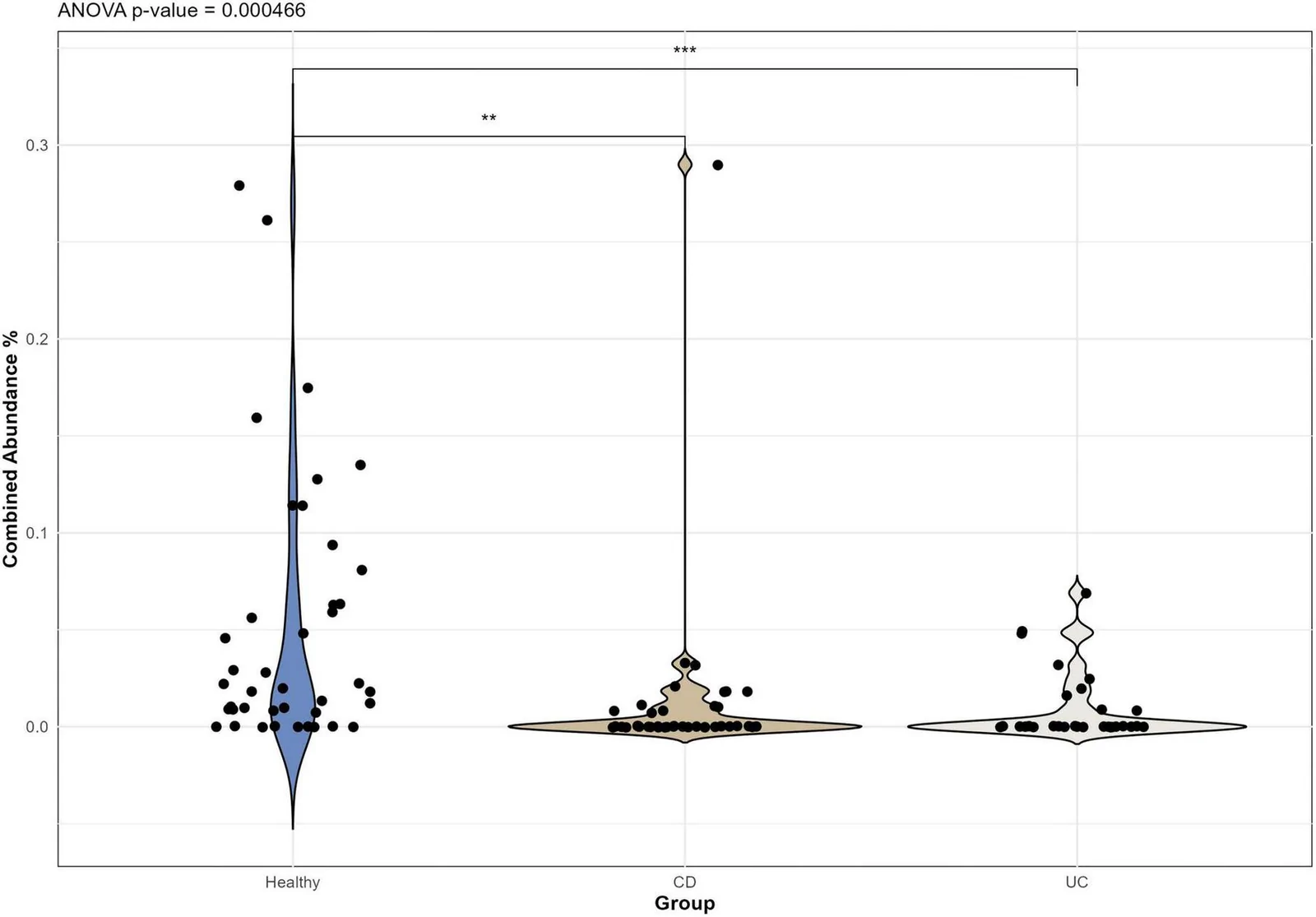
Distribution of the combined relative abundance of the four members from the *Lachnospiraceae* family across groups. A one-way ANOVA yielded an overall. *P* = 0.000466. **P < 0.01, ***P < 0.001.

Based on the metabolomics validation of bilirubin metabolites and metagenomics data, we generated a correlation network (Figure 2). A total of 472 bacterial species were found to correlate with at least one of the bilirubin metabolites shown in Figure 5, and for 30 of these, we observed significant changes in their abundance between patients and the HC group. Notably, our correlation analyses revealed strong associations between members of the *Lachnospiraceae* family (*Blautia* sp. SG-772, *Lachnospiraceae* sp. UBA4711, UBA5902, UBA6452), and the bilirubin metabolites I-urobilinogen, stercobilinogen, and stercobilin (Figure 7).

## 4 Discussion

### 4.1 Metagenomics profile

Our metagenomic findings align well with the existing literature on dysbiosis in IBD patients’ microbiomes (Abdelbary et al., 2022). There is a significant reduction in gut bacterial diversity in patients with active IBD, whereas patients in remission exhibit lower diversity than HC (HC vs active CD *p* < 0.01; HC vs active UC *p* < 0.001; Supplementary Figure 1). This suggests microbial dysbiosis is closely linked to disease activity and may recover during remission. Seventy-two bacterial species showed significant abundance changes in IBD patients, likely affecting metabolic functions, such as bilirubin catabolism, that depend on specific bacterial enzymes. This conclusion is strengthened by beta-diversity patterns between UC and CD patients, which differ from those of HC, indicating that despite distinct clinical presentations, these conditions share common disruptions in gut microbial communities.

### 4.2 Metabolomics profile

We conducted both untargeted metabolomic analyses of all samples. Our untargeted metabolomics approach, utilizing the GNPS FBMN workflow and statistical analysis, offers a reliable method for exploring the gut metabolome. The MolNetEnhancer analysis based on the FBMN identified amino acids, peptides, and bile acids as the predominant metabolite classes in stool samples, consistent with established literature (Jagt et al., 2022). However, we did not observe significant differences between the groups in the metabolomic PCA. Nevertheless, we observed more than 100 features that were significantly upregulated or downregulated (FDR < 0.05) when comparing the two patient groups with the HC (Figure 5). This suggests that while the impact of each metabolite alone may not be evident on a larger scale, it highlights the importance of targeted metabolomic strategies for identifying pathway-specific changes that can be obscured in global metabolomic comparisons, particularly when analyzing complex biological matrices such as stool samples. These changes in bilirubin metabolite levels suggest that important enzymatic processes mediated by the microbiota of IBD patients are disrupted.

### 4.3 Bilirubin metabolites

Downstream bilirubin digestion in the body primarily occurs in the gut and is mediated by the gut microbiome. Given its role in inflammation and digestion, along with preliminary results from our group showing a strong decrease in bilirubin metabolite concentrations in mice with IBD, we focused our targeted metabolite validation on bilirubin and its metabolites. Our findings revealed a distinct pattern in the concentrations of bilirubin metabolites among different patient groups, offering insights into altered metabolic pathways in IBDs. The FBMN enabled us to differentiate and accurately identify bilirubin metabolites, validating I-urobilinogen and several related metabolites. These validations enabled us to observe significant differences in the concentrations of D-urobilin, stercobilinogen, and stercobilin between patients with IBDs and HC.

Bilirubin and its reduced downstream metabolite, stercobilinogen, are known for their antioxidant effects (Nakamura et al., 2006; Zhang et al., 2025). The role of stercobilin itself is more complex: comparative *in vitro* assays indicate it retains some radical scavenger similar to bilirubin (Mölzer et al., 2012), but it has also been found to stimulate pro-inflammatory cytokines like TNF-α and IL-1β in macrophages. Additionally, it accumulates in a mouse model of chronic low-grade inflammation (Sanada et al., 2020), indicating that its overall physiological function may depend on the context. This complexity highlights the need for further research. Conversely, urobilin does not have these antioxidant defenses and has been linked to increased adiposity and insulin resistance in humans (Kipp et al., 2023). In IBD patients, lower levels of stercobilinogen and stercobilin may reflect reduced antioxidant capacity within the gut lumen, potentially contributing to the oxidative burden observed in IBD. Furthermore, the consistent changes in these metabolite levels across the UC and CD cohorts suggest a shared metabolic profile, despite differences in clinical and histopathological characteristics. This observation is consistent with our metagenomic findings, which reveal common patterns of microbial dysbiosis, supporting the hypothesis that specific key metabolic disturbances may underlie various IBD phenotypes.

### 4.4 Multi-omics correlations

Integrating metagenomic and metabolomic data through multi-omics correlation analysis revealed intriguing relationships between specific bacterial species and bilirubin metabolites. The recent discovery of BilR as the enzyme that reduces bilirubin to urobilinogen, along with a recent preprint describing a second enzyme, bilirubin vinyl reductase (BilV), that acts alongside BilR to complete this reduction, highlights the importance of our findings and the multi-step, enzymatic process involved in microbial bile pigment breakdown (Hall et al., 2024; Jiang et al., 2026). Similarly, the still-unknown conversion of I-urobilinogen to stercobilinogen may likewise depend on one or more uncharacterized reductases, possibly explaining the altered bilirubin metabolite patterns seen in patient samples. The unchanged levels of I-urobilinogen, alongside significant decreases in downstream metabolites, suggest that microbiome changes mainly impact bacteria involved in the later stages of bilirubin breakdown.

This finding is particularly exciting, as *Lachnospiraceae* are key prominent butyrate-producing bacteria in the human gut, and their absence has been linked to IBD development (Vacca et al., 2020). The observed positive correlation between *Lachnospiraceae* abundance and various bilirubin metabolites suggests that these bacteria may play a crucial role in the later stages of the bilirubin breakdown, potentially containing enzymes that convert I-urobilinogen into stercobilinogen. The reduction in *Lachnospiraceae* observed in IBD patients aligns with the decreased levels of stercobilinogen and stercobilin, further supporting this hypothesis. This link between *Lachnospiraceae* and bilirubin metabolism offers a new perspective on the metabolic functions of this bacterial family, highlighting roles beyond its established role in short-chain fatty acid production.

Our findings have implications beyond just nominating candidate taxa, offering broader insights into mechanisms and potential clinical applications. A recent preprint reported the discovery of bilirubin vinyl reductase (BilV), a second enzyme working alongside BilR to fully reduce bilirubin to urobilinogen (Jiang et al., 2026). This indicates that microbial bile pigment breakdown involves multiple enzymes acting sequentially, rather than a single catalyst. The step converting I-urobilinogen to stercobilinogen most affected in our IBD cohorts may therefore depend on one or more still-unidentified reductases, potentially encoded within *Lachnospiraceae*. Our work expands the enzymatic map of the pathway from urobilinogen production (Hall et al., 2024; Jiang et al., 2026) to its final, most clinically relevant reactions. The depletion of stercobilinogen and stercobilin was consistent across UC and CD suggests these metabolites could serve as non-invasive, stool-based biomarkers of bilirubin pathway dysfunction, aligning with broader efforts to develop fecal metabolomic signatures for IBD detection and monitoring (Adolph et al., 2022; Vich Vila et al., 2024). Additionally, *Lachnospiraceae*, particularly *Blautia*, are increasingly recognized for their anti-inflammatory and potential probiotic effects (Liu et al., 2021). Restoring these taxa through diet, prebiotics, or microbiome-targeted therapies may offer a promising, though still hypothetical, approach to re-establishing normal bilirubin metabolism in the inflamed gut. While these possibilities need further experimental validation, they illustrate how the identified metabolic bottleneck could inform biomarker development and therapeutic strategies for IBD.

### 4.5 Limitations of the study

This study has several limitations to consider. The cross-sectional design does not allow definitive establishment of causal relationships between microbial changes and alterations in bilirubin metabolites. However, the consistency of the observed patterns across both UC and CD cohorts, two clinically distinct conditions, strengthens the biological plausibility of these associations and motivates longitudinal studies tracking patients through disease flares and remission. Our findings have not yet been validated in an independent cohort, and replication in geographically and We ethnically diverse populations will be important to establish generalizability. However, the strong statistical significance and alignment with the recently described BilR pathway provide a robust foundation. We should note that potential confounders such as dietary changes, medication use, and supplement intake may independently influence both the gut microbiome and bilirubin metabolism. The retrospective design limited the ability to collect detailed, long-term medication histories, meaning the observed taxa–metabolite correlations could not be adjusted for medication effects. Since IBD treatments (like 5-ASAs, biologics, and immunomodulators) can alter both microbiome composition and host metabolism, it is possible that medication-driven shifts, rather than the disease itself, contribute to the metabolic bottleneck seen. Clarifying these effects will require future studies with controlled protocols and medication stratification. Finally, the predicted role of *Lachnospiraceae* in the I-urobilinogen to stercobilinogen conversion awaits functional validation through *in vitro* enzyme assays or gnotobiotic models, experiments now enabled by the candidate taxa and metabolic framework identified here.

## 5 Conclusion

In conclusion, our multi-omics analysis reveals that disruption of the bilirubin catabolic pathway is a notable and consistent feature of IBD, with significant reductions in stercobilinogen and stercobilin levels across both UC and CD patients. The strong correlation between *Lachnospiraceae* abundance and these downstream metabolites identifies this bacterial family as a candidate mediator of the I-urobilinogen to stercobilinogen conversion, extending its known functional repertoire beyond short-chain fatty acid production. These findings open new and exciting avenues for biomarker development and for targeted investigation of the enzymatic machinery linking gut microbial ecology to bilirubin metabolism in inflammatory disease.

## Data Availability

The Mass Spectrometry data presented in the study are deposited in the MassIVE repository, accession number MSV000098724. The sequencing data presented in the study are deposited in the ENA repository, accession number PRJEB122858.

## Footnotes

1
http://gnps.ucsd.edu

2
https://github.com/lh3/seqtk

3
https://gtdb.ecogenomic.org

## References

[B1] Abdelbary M. M. H. Hatting M. Bott A. Dahlhausen A. Keller D. Trautwein C. et al. (2022). The oral-gut axis: Salivary and fecal microbiome dysbiosis in patients with inflammatory bowel disease. *Front. Cell Infect. Microbiol*. 12:1010853. 10.3389/fcimb.2022.1010853 36275026 PMC9585322

[B2] Adolph T. E. Meyer M. Schwärzler J. Mayr L. Grabherr F. Tilg H. (2022). The metabolic nature of inflammatory bowel diseases. *Nat. Rev. Gastroenterol. Hepatol*. 19 753–767. 10.1038/s41575-022-00658-y 35906289

[B3] Adusumilli R. Mallick P. (2017). “Data conversion with proteowizard msconvert,” in *Proteomics: Methods and Protocols*, eds Comai L. Katz J. E. Mallick P. (New York, NY: Springer), 339–368. 10.1007/978-1-4939-6747-6_23 28188540

[B4] Aron A. T. Gentry E. C. McPhail K. L. Nothias L. F. Nothias-Esposito M. Bouslimani A. et al. (2020). Reproducible molecular networking of untargeted mass spectrometry data using GNPS. *Nat. Protoc*. 15 1954–1991. 10.1038/s41596-020-0317-5 32405051

[B5] Belkaid Y. Hand T. W. (2014). Role of the microbiota in immunity and inflammation. *Cell* 157 121–141. 10.1016/j.cell.2014.03.011 24679531 PMC4056765

[B6] Blanco A. Blanco G. (2017). “Heme metabolism,” in *Medical Biochemistry*, eds Blanco A. Blanco G. (Boston: Academic Press), 401–412. 10.1016/B978-0-12-803550-4.00017-3

[B7] Boyle N. R. Sekela J. J. Wang M. Mannochio-Russo H. Pyo J. J. Kim M. S. et al. (2026). Glucuronidation metabolomic fingerprinting to map host-microbe metabolism. *Nat. Commun*. 17:6646. 10.1038/s41467-026-73398-1 42161945 PMC13381908

[B8] Canesin G. Hejazi S. M. Swanson K. D. Wegiel B. (2020). Heme-derived metabolic signals dictate immune responses. *Front. Immunol*. 11:66. 10.3389/fimmu.2020.00066 32082323 PMC7005208

[B9] Chen L. A. Li H. Jones D. Wang C. Li D. Radin A. et al. (2024). Unaffected relatives with high IBD polygenic risk scores from multiplex crohn’s disease families carry microbiomes that protect il-10 deficient mice against intestinal inflammation. *Inflammatory. Bowel. Diseases* 30 S69–S70. 10.1093/ibd/izae020.150

[B10] Chen S. Zhou Y. Chen Y. Gu J. (2018). Fastp: An ultra-fast all-in-one FASTQ preprocessor. *Bioinformatics* 34 i884–i890. 10.1093/bioinformatics/bty560 30423086 PMC6129281

[B11] Dührkop K. Fleischauer M. Ludwig M. Aksenov A. A. Melnik A. V. Meusel M. et al. (2019). SIRIUS 4: A rapid tool for turning tandem mass spectra into metabolite structure information. *Nat. Methods* 16 299–302. 10.1038/s41592-019-0344-8 30886413

[B12] Engelking L. R. (2015). “Heme degradation,” in *Textbook of Veterinary Physiological Chemistry*, 3rd Edn, ed. Engelking L. R. (Boston: Academic Press), 202–207. 10.1016/B978-0-12-391909-0.50033-5

[B13] Ernst M. Kang K. B. Caraballo-Rodríguez A. M. Nothias L. F. Wandy J. Chen C. et al. (2019). Mol net enhancer: Enhanced molecular networks by integrating metabolome mining and annotation tools. *Metabolites* 9:144. 10.3390/metabo9070144 31315242 PMC6680503

[B14] Fiehn O. Robertson D. Griffin J. van der Werf M. Nikolau B. Morrison N. et al. (2007). The metabolomics standards initiative (MSI). *Metabolomics* 3 175–178. 10.1007/s11306-007-0070-6

[B15] Fogelson K. A. Dorrestein P. C. Zarrinpar A. Knight R. (2023). The gut microbial bile acid modulation and its relevance to digestive health and diseases. *Gastroenterology* 164 1069–1085. 10.1053/j.gastro.2023.02.022 36841488 PMC10205675

[B16] Foster J. A. McVey Neufeld K. A. (2013). Gut-brain axis: How the microbiome influences anxiety and depression. *Trends. Neurosci*. 36 305–312. 10.1016/j.tins.2013.01.005 23384445

[B17] Gregor R. Probst M. Eyal S. Aksenov A. Sasson G. Horovitz I. et al. (2022). Mammalian gut metabolomes mirror microbiome composition and host phylogeny. *ISME J*. 16 1262–1274. 10.1038/s41396-021-01152-0 34903850 PMC9038745

[B18] Haag L. M. Siegmund B. (2015). Intestinal microbiota and the innate immune system - A crosstalk in crohn’s disease pathogenesis. *Front. Immunol*. 6:489. 10.3389/fimmu.2015.00489 26441993 PMC4585200

[B19] Hall B. Levy S. Dufault-Thompson K. Arp G. Zhong A. Ndjite G. M. et al. (2024). BILR is a gut microbial enzyme that reduces bilirubin to urobilinogen. *Nat. Microbiol*. 9 173–184. 10.1038/s41564-023-01549-x 38172624 PMC10769871

[B20] Jacobs J. P. Goudarzi M. Singh N. Tong M. McHardy I. H. Ruegger P. et al. (2016). A disease-associated microbial and metabolomics state in relatives of pediatric inflammatory bowel disease patients. *Cell. Mol. Gastroenterol. Hepatol*. 2 750–766. 10.1016/j.jcmgh.2016.06.004 28174747 PMC5247316

[B21] Jagt J. Z. Verburgt C. M. de Vries R. de Boer N. K. H. Benninga M. A. de Jonge W. J. et al. (2022). Faecal metabolomics in paediatric inflammatory bowel disease: A systematic review. *J. Crohns. Colitis*. 16 1777–1790. 10.1093/ecco-jcc/jjac079 35679608 PMC9683079

[B22] Jiang A. K. Grant M. R. Arp G. Dufault-Thompson K. Clarke A. M. Li Y. et al. (2026). *Discovery Of Bilv Reveals A Multienzymatic Basis For Bilirubin Reduction Across Vertebrate Gut Microbiomes.* BIORXIV. 10.64898/2026.06.01.729425

[B23] Kipp Z. A. Xu M. Bates E. A. Lee W. H. Kern P. A. Hinds T. D. (2023). Bilirubin levels are negatively correlated with adiposity in obese men and women, and its catabolized product. *Antioxidants* 12:170. 10.3390/antiox12010170 36671031 PMC9854555

[B24] Kumar A. Al-Hassi H. O. Steed H. Phipps O. Brookes M. J. (2022). Bile acids and the microbiome: Making sense of this dynamic relationship in their role and management in crohn’s disease. *Can. J. Gastroenterol. Hepatol*. 2022:8416578. 10.1155/2022/8416578 35360442 PMC8964223

[B25] Langmead B. Salzberg S. L. (2012). Fast gapped-read alignment with Bowtie 2. *Nat. Methods*. 9 357–359. 10.1038/nmeth.1923 22388286 PMC3322381

[B26] Lee M. H. Nuccio S. P. Mohanty I. Hagey L. R. Dorrestein P. C. Chu H. et al. (2024). How bile acids and the microbiota interact to shape host immunity. *Nat. Rev. Immunol*. 24 798–809. 10.1038/s41577-024-01057-x 39009868

[B27] Levhar N. Hadar R. Braun T. Shacham H. Algavi Y. Naamneh R. et al. (2026). Fecal metabolic signals are associated with changes in microbiota and systemic metabolic pathways in crohn’s disease. *Sci. Rep*. 16:6991. 10.1038/s41598-026-38558-9 41629500 PMC12920629

[B28] Li H. Handsaker B. Wysoker A. Fennell T. Ruan J. Homer N. et al. (2009). The sequence alignment/map format and SAMtools. *Bioinformatics*. 25 2078–2079. 10.1093/bioinformatics/btp352 19505943 PMC2723002

[B29] Liu X. Mao B. Gu J. Wu J. Cui S. Wang G. et al. (2021). Blautia-a new functional genus with potential probiotic properties? *Gut. Microbes*. 13 1–21. 10.1080/19490976.2021.1875796 33525961 PMC7872077

[B30] Lu J. Breitwieser F. P. Thielen P. Salzberg S. L. (2017). Bracken: Estimating species abundance in metagenomics data. *Peer. J. Comput. Sci*. 3:e104. 10.7717/peerj-cs.104 40271438 PMC12016282

[B31] Ma X. Lu X. Zhang W. Yang L. Wang D. Xu J. et al. (2022). Gut microbiota in the early stage of crohn’s disease has unique characteristics. *Gut. Pathog*. 14:46. 10.1186/s13099-022-00521-0 36517872 PMC9753350

[B32] Mohanty I. Allaband C. Mannochio-Russo H. El Abiead Y. Hagey L. R. Knight R. et al. (2024). The changing metabolic landscape of bile acids - keys to metabolism and immune regulation. *Nat. Rev. Gastroenterol. Hepatol*. 21 493–516. 10.1038/s41575-024-00914-3 38575682 PMC12248421

[B33] Mölder F. Jablonski K. P. Letcher B. Hall M. B. van Dyken P. C. Tomkins-Tinch C. H. et al. (2021). Sustainable data analysis with snakemake. *F. 1000 Res*. 10:33. 10.12688/f1000research.29032.3 34035898 PMC8114187

[B34] Mölzer C. Huber H. Steyrer A. Ziesel G. Ertl A. Plavotic A. et al. (2012). In vitro antioxidant capacity and antigenotoxic properties of protoporphyrin and structurally related tetrapyrroles. *Free. Radic. Res*. 46 1369–1377. 10.3109/10715762.2012.715371 22861140

[B35] Mölzer C. Wallner M. Kern C. Tosevska A. Zadnikar R. Doberer D. et al. (2017). Characteristics of the heme catabolic pathway in mild unconjugated hyperbilirubinemia and their associations with inflammation and disease prevention. *Sci. Rep*. 7:755. 10.1038/s41598-017-00933-y 28389660 PMC5429724

[B36] Nakamura T. Sato K. Akiba M. Ohnishi M. (2006). Urobilinogen, as a Bile Pigment Metabolite. *Antioxidant. Function J. Oleo. Sci.* 55 191–197. 10.5650/jos.55.191

[B37] Nothias L. F. Petras D. Schmid R. Dührkop K. Rainer J. Sarvepalli A. et al. (2020). Feature-based molecular networking in the GNPS analysis environment. *Nat. Methods*. 17 905–908. 10.1038/s41592-020-0933-6 32839597 PMC7885687

[B38] Rapozo D. C. Bernardazzi C. de Souza H. S. (2017). Diet and microbiota in inflammatory bowel disease: The gut in disharmony. *World J. Gastroenterol*. 23 2124–2140. 10.3748/wjg.v23.i12.2124 28405140 PMC5374124

[B39] Rashed R. Valcheva R. Dieleman L. A. (2022). Manipulation of gut Microbiota as a Key Target for Crohn’s Disease. *Front. Med* . 9:887044. 10.3389/fmed.2022.887044 35783604 PMC9244564

[B40] Sanada S. Suzuki T. Nagata A. Hashidume T. Yoshikawa Y. Miyoshi N. (2020). Intestinal microbial metabolite stercobilin involvement in the chronic inflammation of ob/ob mice. *Sci. Rep*. 10:6479. 10.1038/s41598-020-63627-y 32296105 PMC7160104

[B41] Schmid R. Heuckeroth S. Korf A. Smirnov A. Myers O. Dyrlund T. S. et al. (2023). Integrative analysis of multimodal mass spectrometry data in MZmine 3. *Nat. Biotechnol*. 41 447–449. 10.1038/s41587-023-01690-2 36859716 PMC10496610

[B42] Vacca M. Celano G. Calabrese F. M. Portincasa P. Gobbetti M. De Angelis M. (2020). The controversial role of human gut lachnospiraceae. *Microorganisms* 8:573. 10.3390/microorganisms8040573 32326636 PMC7232163

[B43] Vich Vila A. Zhang J. Liu M. Faber K. N. Weersma R. K. (2024). Untargeted faecal metabolomics for the discovery of biomarkers and treatment targets for inflammatory bowel diseases. *Gut*. 73 1909–1920. 10.1136/gutjnl-2023-329969 39002973 PMC11503092

[B44] Wang M. Carver J. J. Phelan V. V. Sanchez L. M. Garg N. Peng Y. et al. (2016). Sharing and community curation of mass spectrometry data with global natural products social molecular networking. *Nat. Biotechnol.* 34 828–837. 10.1038/nbt.3597 27504778 PMC5321674

[B45] Wood D. E. Lu J. Langmead B. (2019). Improved metagenomic analysis with Kraken 2. *Genome. Biol*. 20:257. 10.1186/s13059-019-1891-0 31779668 PMC6883579

[B46] Wright E. K. Kamm M. A. Teo S. M. Inouye M. Wagner J. Kirkwood C. D. (2015). Recent advances in characterizing the gastrointestinal microbiome in crohn’s disease: a systematic review. *Inflamm. Bowel. Dis*. 21 1219–1228. 10.1097/MIB.0000000000000382 25844959 PMC4450900

[B47] Xue M. Lee S. H. Shao J. Leibovitzh H. Huynh H. Q. Griffiths A. M. et al. (2025). Metabolomics reveal distinct molecular pathways associated with future risk of crohn’s Disease. *Gut. Microbes*. 17:2546998. 10.1080/19490976.2025.2546998 40910526 PMC12416195

[B48] Zhang Y. Luan H. Song P. (2025). Bilirubin metabolism and its application in disease prevention: Mechanisms and research advances. *Inflamm. Res*. 74:81. 10.1007/s00011-025-02049-w 40413269

[B49] Zoroddu S. Di Lorenzo B. Paliogiannis P. Mangoni A. A. Carru C. Zinellu A. (2024). The association between bilirubin concentrations and inflammatory bowel disease: Insights from a systematic review and meta-analysis. *Eur. J. Clin. Invest*. 54:e14281. 10.1111/eci.14281 38970234

