# Supplementary material for: Disrupted terminal bilirubin catabolism links Lachnospiraceae depletion to inflammatory bowel disease
Source: Front Microbiol. 2026 Aug 17;17:1871609. doi: 10.3389/fmicb.2026.1871609 (PMC13526984; doi:10.3389/fmicb.2026.1871609)
Supplement: Supplementary file 1 [file Data_Sheet_1.pdf]

Supporting information

Disrupted Terminal Bilirubin Catabolism Links Lachnospiraceae Depletion to Inflammatory Bowel Disease

Omer Sabti[a], Ksenia Lialin Tzadikov[a], Valeria Ivanova[b], Mally Dori-Bachash[b], Shaked Uzi-Gavrilov[a], Zohar Tik[a], Roi Mashiach[a], Alvah Zorea[d], Itzhak Mizrahi[d], Arik Segal[e], Keren Moyal-Attias[e], Eran Elinav[b,c], and Michael M. Meijler*[a]

[a]Department of Chemistry, Ben Gurion University of the Negev, Be'er Sheva, Israel; [b]Department of Systems Immunology, Weizmann Institute of Science, Rehovot, Israel; [c]Division of Microbiome & Cancer, German Cancer Research Center (DKFZ), Heidelberg, Germany; [d]Department of Life Sciences, Ben Gurion University of the Negev, Be'er Sheva, Israel; [e]Department of Gastroenterology, Soroka University Medical Center, Be'er Sheva, Israel;

*Department of Chemistry, Ben Gurion University of the Negev, Be'er Sheva 84105, Israel, E-mail: meijler@bgu.ac.il.

Abstract: Molecular links between gut microbiota dynamics and host health in Inflammatory Bowel Diseases (IBD), particularly those involving the bilirubin metabolic pathway, are critical for understanding disease pathophysiology. Gut microbiome imbalances are known to affect IBD. Since bilirubin and its derivatives have antioxidant properties, microbial disruption of their metabolism could exacerbate intestinal inflammation. A multi-omics approach was used to identify metabolic changes, analyzing fecal samples from 119 IBD patients (Crohn's disease and ulcerative colitis) and healthy controls via UPLC/HRMS metabolomics and shotgun metagenomic sequencing. The results revealed significantly lower levels of the bilirubin metabolites stercobilinogen and stercobilin in IBD patients, alongside reduced gut bacterial diversity in active disease. A strong positive correlation was identified between the abundance of *Lachnospiraceae* bacteria and key bilirubin metabolites. Our findings suggest that disruption of the bilirubin catabolic pathway is a key feature in IBD pathophysiology, highlighting a novel metabolic role for *Lachnospiraceae* and potentially identifying new biomarkers for disease activity.

Keywords: Bilirubin • IBD • Microbiota • Metabolomics • Microbial Chemical Communication

Table of Contents

Data availability 2
Study Population 3
Cohort Characteristics .... **Error! Bookmark not defined.**
Sample Collection .... **Error! Bookmark not defined.**
Metabolomics .... **Error! Bookmark not defined.**
Metabolite Extraction .... **Error! Bookmark not defined.**
UHPLC-HRMS/MS analysis .... **Error! Bookmark not defined.**
Data Processing .... **Error! Bookmark not defined.**
I-urobilinogen validation .... **Error! Bookmark not defined.**
Metagenomics .... **Error! Bookmark not defined.**
DNA Purification .... **Error! Bookmark not defined.**
Shotgun Sequencing .... **Error! Bookmark not defined.**
Data Processing .... **Error! Bookmark not defined.**
Supporting Figures and Tables .... **Error! Bookmark not defined.**
Table S1 4
Figure S1. 6
Figure S2. 7
Figure S3. 7
Figure S4. 8
Figure S5. 8
Figure S6. 9
Figure S7. 9
Figure S8. 10
Figure S9. .... **Error! Bookmark not defined.**

Data availability

All the mass spectrometry data used in the preparation of this manuscript are publicly available at the MassIVE repository at the UCSD Center for Computational Mass Spectrometry website (massive.ucsd.edu). ftp://MSV000098724@massive-ftp.ucsd.edu
Username: MSV000098724_reviewer, Password: mpO6831kPsg8Y1u2

All code used in the preparation of this manuscript is publicly available at the GitHub repository OmSa203/IBD-Bilirubin-Catabolic-Pathway. For more detailed information, please read the README file.

Category	Characteristic	HC (n=49)	UC (n=31)	CD (n=39)	P-value
**Demographics**	Age (years; mean ± SD)	34.2 ± 9.8	36.5 ± 11.2	36.8 ± 10.5	0.42
	Age range	19-58	22-70	20-65	-
	Male n (%)	25 (51.0)	16 (51.6)	13 (33.3)	0.18
	Female n (%)	24 (49.0)	15 (48.4)	26 (66.7)	0.78
**Clinical Characteristics**	Disease Duration (years; mean ± SD)	N/A	8.2 ± 6.4	9.1 ± 7.2	0.56
	Current Smoker n (%)	7 (14.3)	3 (9.7)	5 (12.8)	0.84
	Former Smoker n (%)	5 (10.2)	4 (12.9)	6 (15.4)	-
	Never Smoker n (%)	37 (75.5)	24 (77.4)	28 (71.8)	-
**Disease Activity**	Remission n/n-with-data(%)	N/A	13/24 (54.5)	7/38 (18.4)	0.03
	Calprotectin µg/g (mean ± SD)	N/A	285 ± 156	312 ± 178	0.48
**Dietary Habits**	Special Diet n (%)	12 (24.5)	15 (48.4)	22 (56.4)	<0.01
	Gluten-free n (%)	3 (6.1)	5 (16.1)	8 (20.5)	0.03
	Lactose-free n (%)	4 (8.2)	6 (19.4)	9 (23.1)	0.02
	Other dietary restrictions n (%)	5 (10.2)	4 (12.9)	5 (12.8)	0.89
**Supplements**	Any Dietary Supplements n (%)	15 (30.6)	18 (58.1)	25 (64.1)	<0.01
	Vitamin D n (%)	8 (16.3)	12 (38.7)	15 (38.5)	0.02
	Iron n (%)	3 (6.1)	8 (25.8)	12 (30.8)	<0.01
	Multiple vitamins n (%)	7 (14.3)	9 (29.0)	11 (28.2)	0.15
**Medications**	Any IBD medication n (%)	N/A	28 (90.3)	35 (89.7)	0.93
	Biologics n (%)	N/A	12 (38.7)	18 (46.2)	0.54
	Immunomodulators n (%)	N/A	15 (48.4)	21 (53.8)	0.65
	5-ASA n (%)	N/A	22 (71.0)	15 (38.5)	<0.01

Table S1 Demographic and Clinical Characteristics. Data presented as mean ± standard deviation. N/A: Not applicable. P-values were calculated using the Chi-square test for categorical variables and Student's t-test or ANOVA for continuous variables as appropriate. Extended clinical data are available in **Table S1a**.

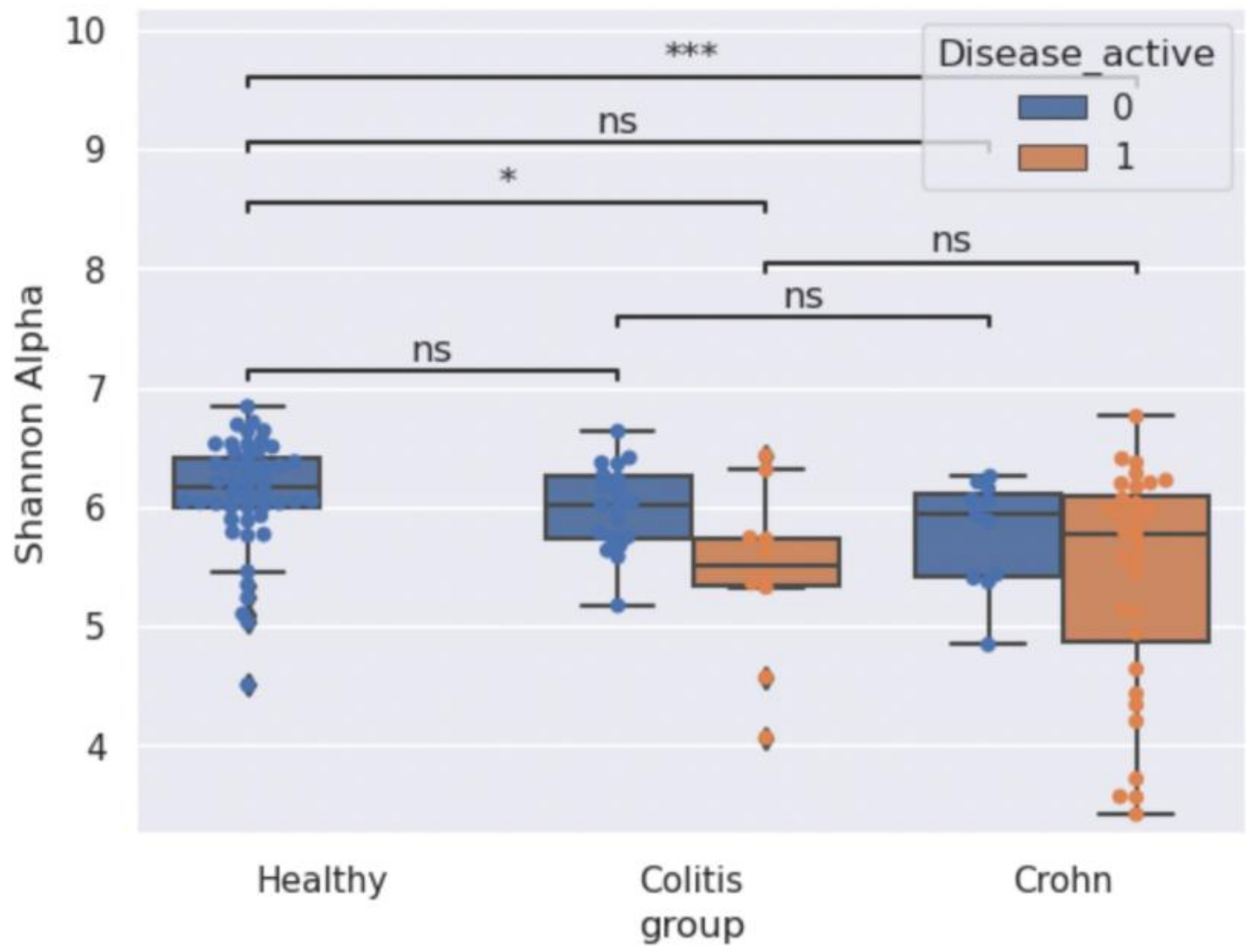

Figure S1**.**

Shannon alpha index based on disease status. *p<0.05, ***p<0.001.

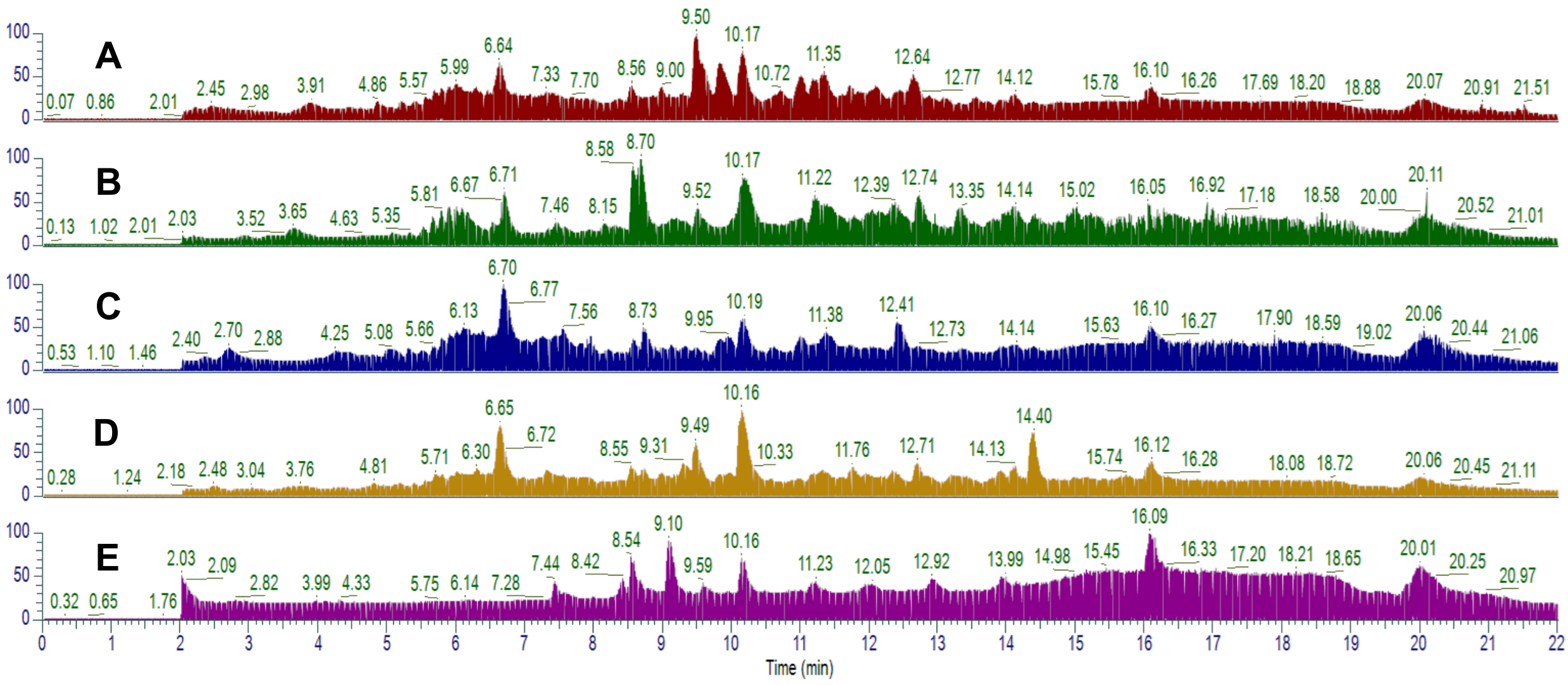

Figure S2.

An example of a total ion chromatogram of samples from the three groups, QC, and the solvent runs; a) sample from the healthy group; b) sample from the ulcerative colitis patient; c) Sample from the Crohn's disease patient; d) mix from all the samples as quality control- QC; e) sample from the solvents as negative control.

I-urobilinogen validation

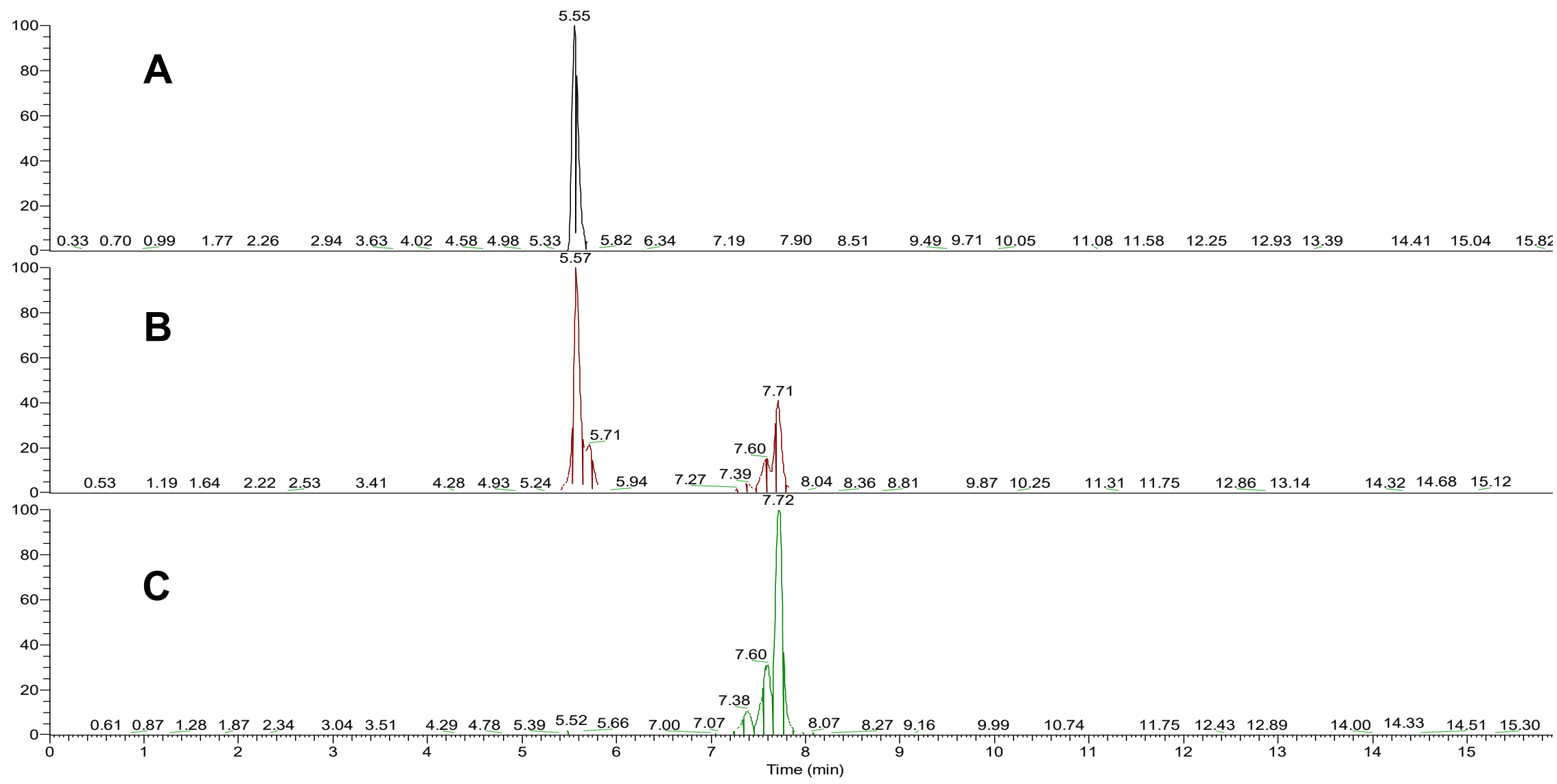

Figure S3.

I-urobilinogen chromatograms. A) mix sample (i.e., QC). Peak at Rt 5.55 min; B) Standard after five days with exposure to oxygen. Peaks at Rt 5.57 min and 7.71 min. C) Standard without exposure to oxygen. Peak at Rt 7.66 min.

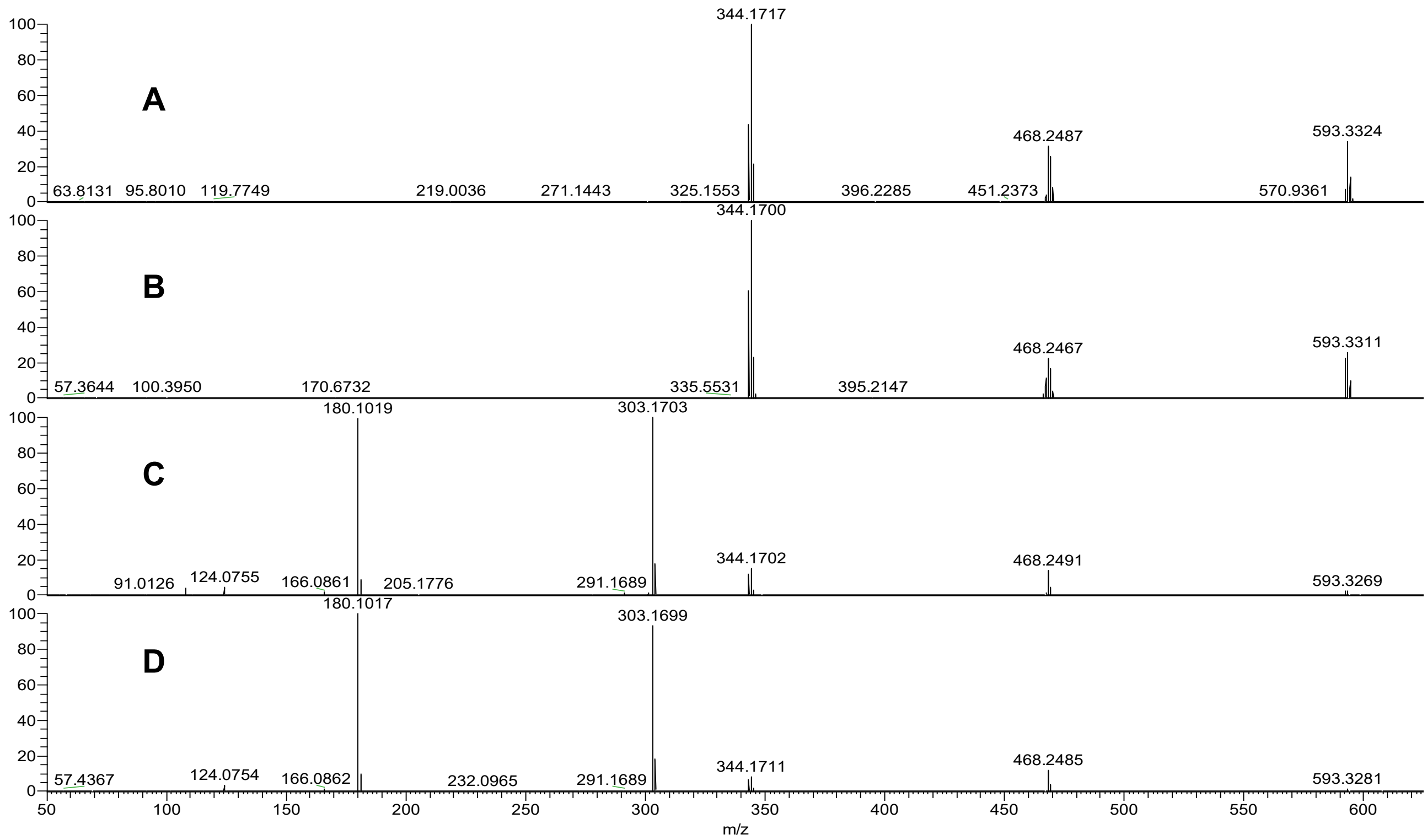

Figure S4.

MS^2 spectra for the chromatogram peaks shown in Figure S2. A) mix sample (i.e., QC). Peak at Rt 5.55 min. B) Standard after five days with exposure to oxygen. Peak at Rt 5.57 min c) Standard after five days with exposure to oxygen. Peak at Rt 7.71 min D) Standard without exposure to oxygen. Peak at Rt 7.66 min.

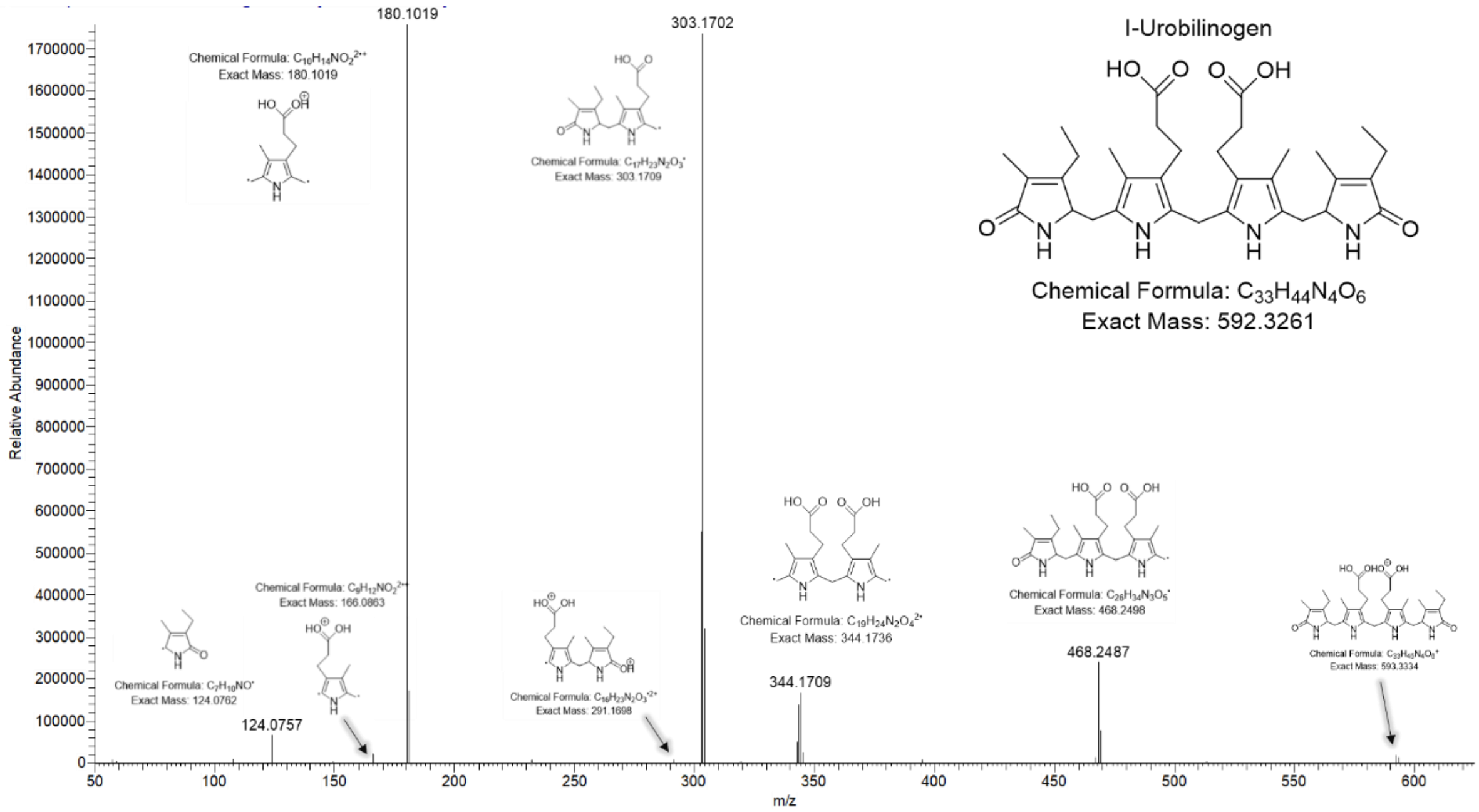

Figure S5**.**

Identification of all fragment peaks accompanying the parent compound with 593.3334m/z. The detection was performed manually and corroborated by SIRIUS predictions.

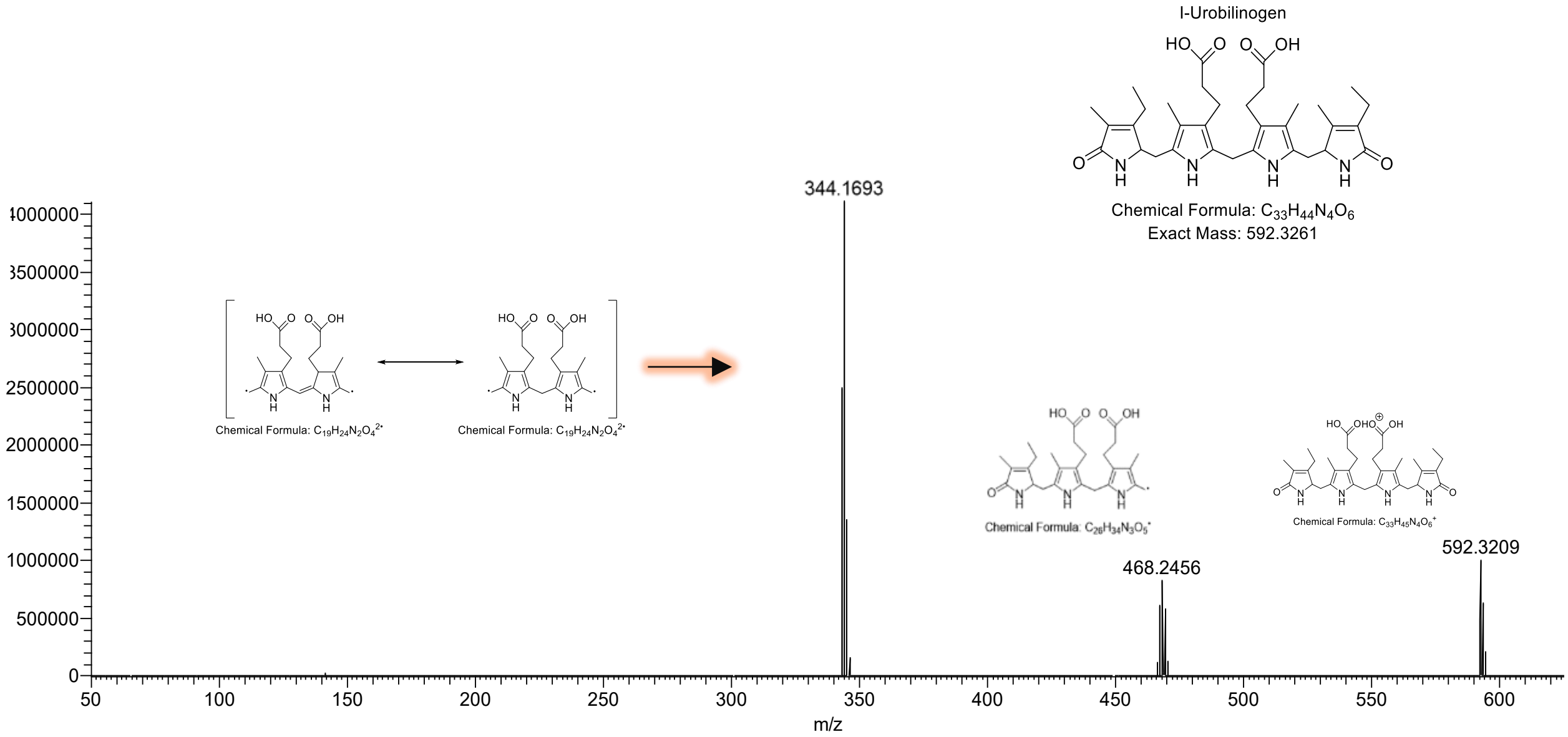

Figure S6**.**

MS^2 spectrum of I-urobilinogen double-bond shifting.

Metagenomics analysis

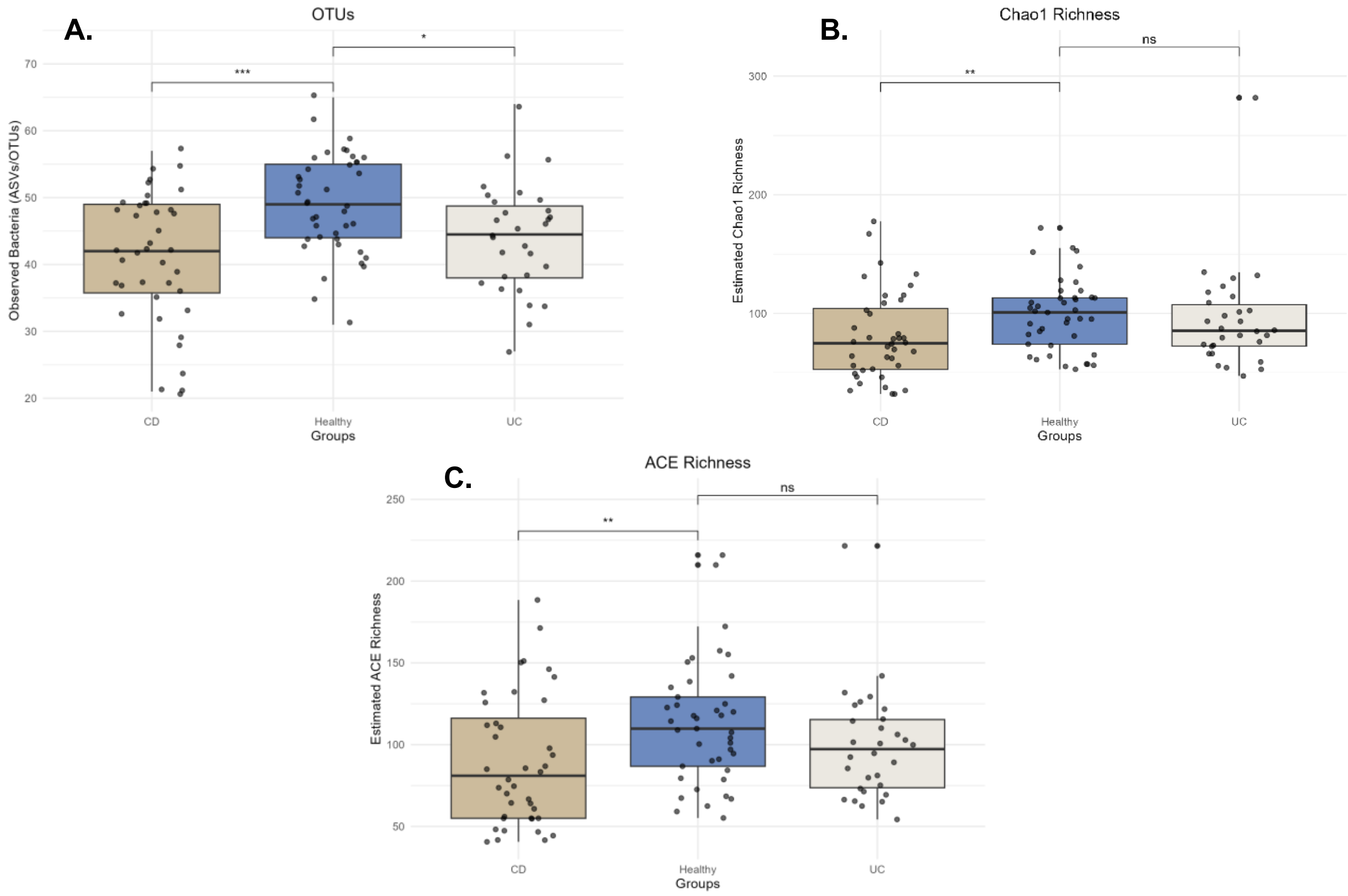

Figure S7**.**

Distribution of richness metrics: A) Observed ASVs. B) Chao1 index. C) ACE index. Each point represents an individual sample. *p<0.05, **p<0.01, ***p<0.001.

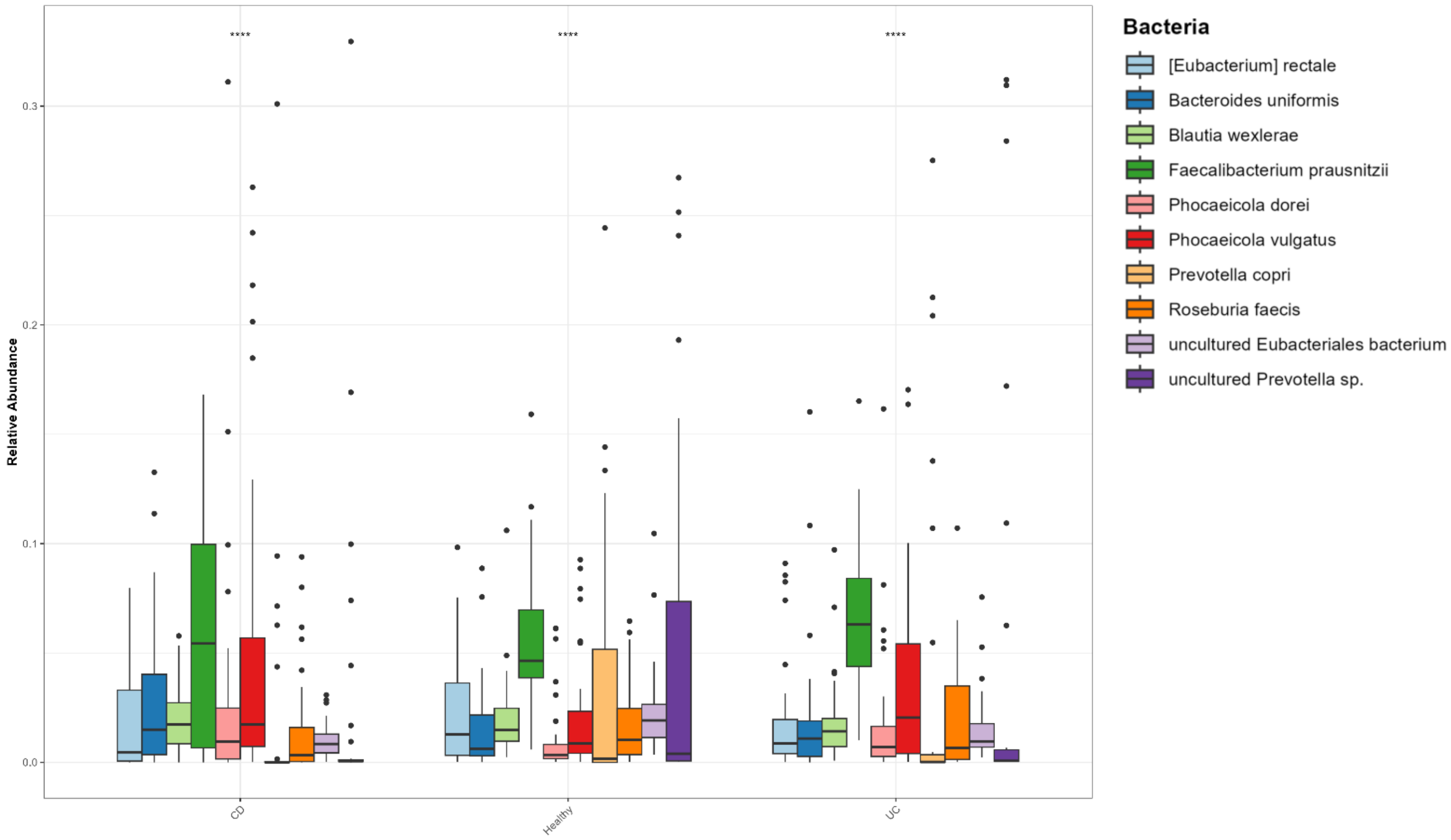

Figure S8**.**

Top 10 most abundant bacterial species. Each boxplot illustrates the relative abundance of a specific bacterial species within the different groups. ****p<0.0001

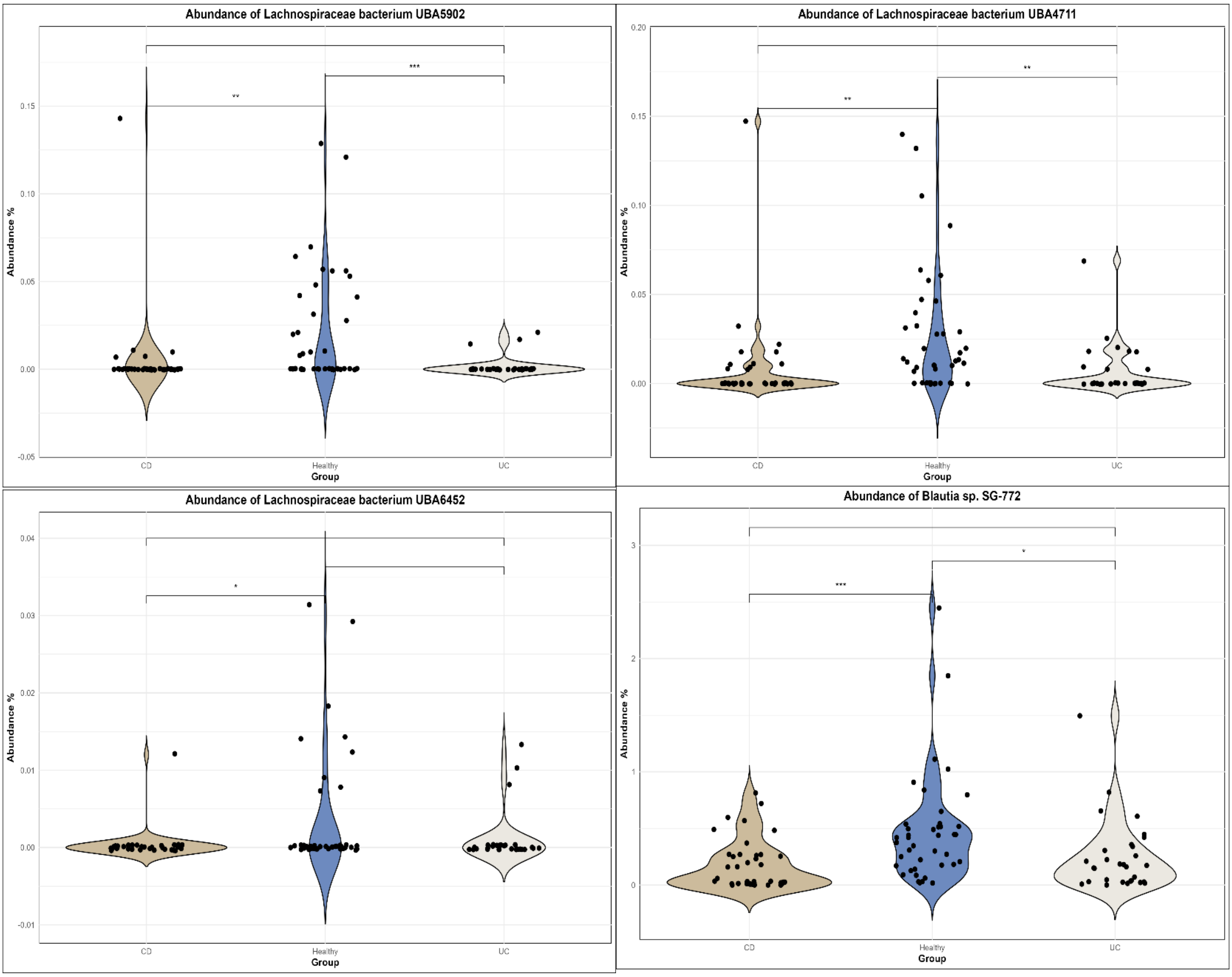

Figure S9. The differential abundance of Lachospiraceae taxon members correlates with the I–urobilinogen–stercobilin pathway across patient groups. *p < 0.05, **p < 0.01, ***p < 0.001.
